# Association of polygenic scores for autism with volumetric MRI phenotypes in cerebellum and brainstem in adults

**DOI:** 10.1186/s13229-024-00611-7

**Published:** 2024-08-07

**Authors:** Salahuddin Mohammad, Mélissa Gentreau, Manon Dubol, Gull Rukh, Jessica Mwinyi, Helgi B. Schiöth

**Affiliations:** 1 Functional Pharmacology and Neuroscience Unit, Department of Surgical Sciences, Uppsala University, Uppsala, Sweden; 2 Department of Women’s and Children’s Health, Science for Life Laboratory, Uppsala University, Uppsala, Sweden

**Keywords:** Autism, Polygenic risk score, Brain MRI, Cerebellum, Brainstem

## Abstract

Previous research on autism spectrum disorders (ASD) have showed important volumetric alterations in the cerebellum and brainstem. Most of these studies are however limited to case-control studies with small clinical samples and including mainly children or adolescents. Herein, we aimed to explore the association between the cumulative genetic load (polygenic risk score, PRS) for ASD and volumetric alterations in the cerebellum and brainstem, as well as global brain tissue volumes of the brain among adults at the population level. We utilized the latest genome-wide association study of ASD by the Psychiatric Genetics Consortium (18,381 cases, 27,969 controls) and constructed the ASD PRS in an independent cohort, the UK Biobank. Regression analyses controlled for multiple comparisons with the false-discovery rate (FDR) at 5% were performed to investigate the association between ASD PRS and forty-four brain magnetic resonance imaging (MRI) phenotypes among ~ 31,000 participants. Primary analyses included sixteen MRI phenotypes: total volumes of the brain, cerebrospinal fluid (CSF), grey matter (GM), white matter (WM), GM of whole cerebellum, brainstem, and ten regions of the cerebellum (I_IV, V, VI, VIIb, VIIIa, VIIIb, IX, X, CrusI and CrusII). Secondary analyses included twenty-eight MRI phenotypes: the sub-regional volumes of cerebellum including the GM of the vermis and both left and right lobules of each cerebellar region. ASD PRS were significantly associated with the volumes of seven brain areas, whereby higher PRS were associated to reduced volumes of the whole brain, WM, brainstem, and cerebellar regions I-IV, IX, and X, and an increased volume of the CSF. Three sub-regional volumes including the left cerebellar lobule I-IV, cerebellar vermes VIIIb, and X were significantly and negatively associated with ASD PRS. The study highlights a substantial connection between susceptibility to ASD, its underlying genetic etiology, and neuroanatomical alterations of the adult brain.

**Supplementary Information:**

The online version contains supplementary material available at 10.1186/s13229-024-00611-7.

## Introduction

Autism spectrum disorder (ASD) is a complex neurodevelopmental disorder characterized by difficulties in social communication, restricted interests, and repetitive behaviors [1]. Many observation about neuroanatomical differences in ASD have been identified using magnetic resonance imaging (MRI) [2–4]. In particular, the role of the cerebellum in social cognition and the pathophysiology of ASD has been well-established [5, 6]. ASD-related genes co-expressed at the cerebellum in the developing brain have been observed to contribute directly to the pathogenesis of ASD [7]. Cerebellar grey matter (GM) volumes correlate with core ASD symptoms [8]. Beyond the core symptoms, the various associated symptoms of ASD affiliate with the disruption in the brainstem [9, 10]. The relationship of ASD with MRI volumetric changes in the cerebellum and brainstem in a genetic context is still underexplored at the adult population level.

ASD is highly genetically heterogeneous and affects ~ 2% of children globally [11]. The genetic liability for ASD influences structural and functional changes in brain anatomy [12, 13], and neuroimaging genetics of ASD has been proposed as a potential novel biomarker for the early diagnosis of ASD [14]. The significant genetic influence in ASD is evidenced by the heritability estimates ranging from 70 to 90% [11, 15], and single nucleotide polymorphism (SNP) based heritability of 11% [16]. Polygenic risk scores (PRS) allow taking into account the cumulative totals of susceptible SNPs that are associated with specific target traits, with each SNP assigned a weight based on its effect size in association with the trait determined by genome-wide association studies (GWAS) [17]. The latest GWAS on ASD enables the construction of ASD PRS that have been effectively explored in association with neuroanatomical changes such as cortical thickness [18, 19], surface area, gyrification [19], and grey matter concentration [20].

A recent meta-analysis on cerebellar volumetric changes in ASD reported a weak but significant (uncorrected) larger global cerebellar volume, and vermal lobules I–V, VI–VII to be most reported with changes in ASD [21]. A population-based cohort addressing children reported higher levels of autistic traits that were inversely associated with cerebellar cortex volumes [22]. Contrarily, decreases in the left cerebellum [23, 24] and reduced cerebellar volume [25, 26] were also reported. In recent studies, no significant differences in the cerebellum were reported exploring a European cohort (274 individuals with ASD and 219 control) [27] and, examination of cerebellar grey-matter volume in children with neurodevelopmental disorders revealed no significant differences in volume of the cerebellar lobules in ASD compared to controls [28]. While the findings are inconclusive, the MRI volumetric studies of ASD are mostly based on children or adolescents [2–4, 28], and brain changes associated with age have been widely observed in ASD [3, 4]. One of the consistent findings in ASD is an accelerated brain growth in early childhood and deceleration in late childhood and adolescence [29–31].

In the last ~ 15 years, there have been hardly any studies published investigating the cerebellar, or brainstem volumetric changes in association with ASD among adults [3, 4, 10]. Recently, Yang et al. (2016) quantitatively estimated regional GM volume abnormalities in adults (18–60 years) with ASD using a meta-analytical approach. The authors found reduced GM volume in the cerebellum with a significant association of age, sex, and intelligence quotient (IQ) [32]. In a longitudinal study by Lange et al. (2015), cerebral development at the whole-brain and the regional levels was compared between individuals with ASD and controls (ages 6–35 years), highlighting a significant difference in growth curves with atypical volume decline in ASD [33].

While previous studies have considerably advanced our understanding of the association between ASD and cerebellar and brainstem volumetric alterations, these studies included neuroimaging data primarily focused on children or adolescents, and were limited mostly to case-control studies. The genetic liability for ASD in association with neuroanatomical changes across the general adult population remains underexplored. Moreover, an emerging perspective postulates that ASD may manifest along a continuum, with a normal distribution of autistic traits evident in the general population, challenging the traditional binary approach of diagnosis in psychiatry research, by recognizing potential intermediate outcomes [34, 35]. This approach is highly suitable for bridging the research gap regarding the connection between the genetic predisposition to ASD and the volumetric measurement of the cerebellum and brainstem among adults in the general population.

**Fig. 1:**
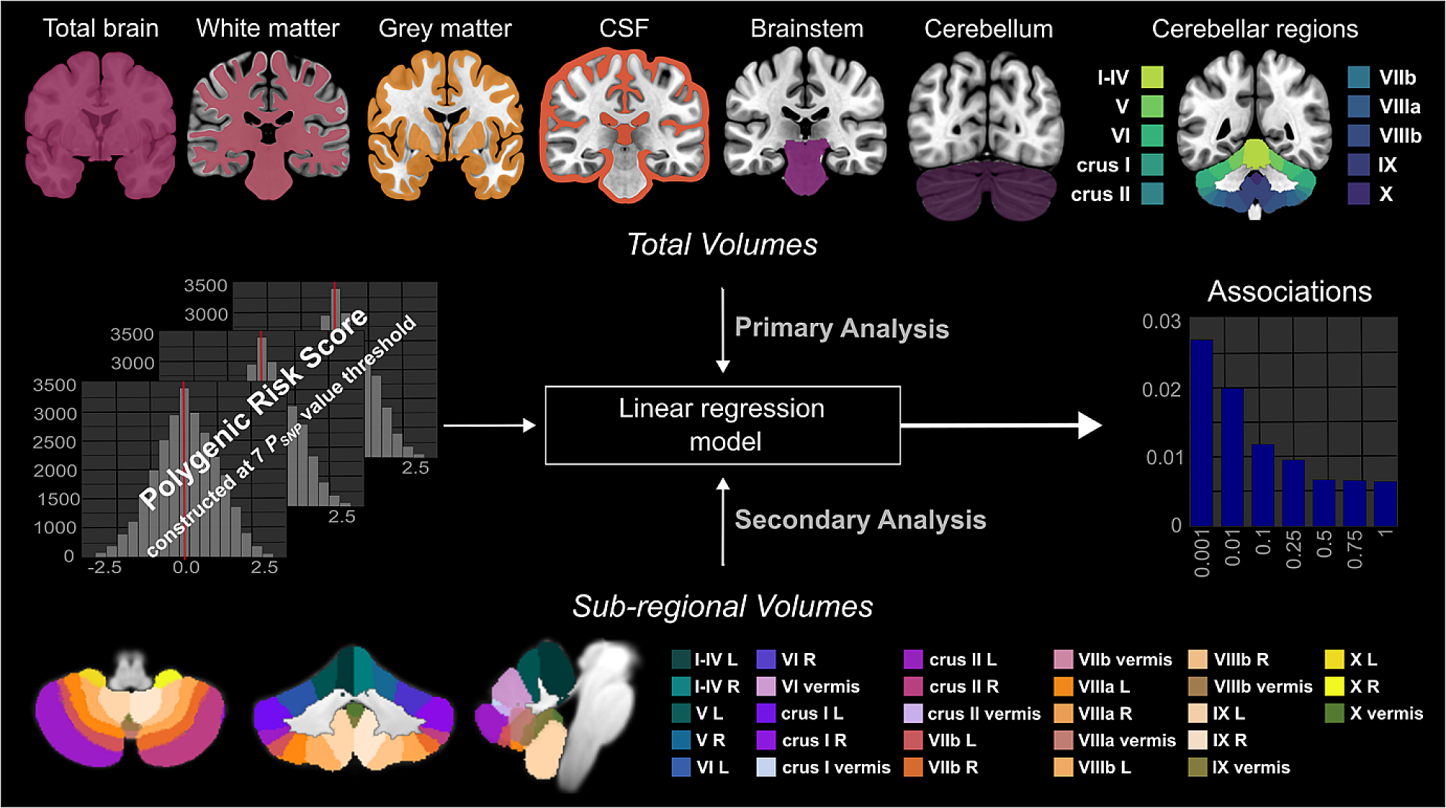
Schematic summary of the study. We investigated the association between ASD PRS and forty-four brain magnetic resonance imaging (MRI) phenotypes among ~ 31,000 participants in the UK Biobank for whom quality-controlled data were available (Details in Supplementary Fig. 1). Primary analyses included sixteen MRI phenotype - total volumes of the brain, CSF, GM, WM, GM of whole cerebellum, brainstem and ten regions of the cerebellum (I_IV, V, VI, VIIb, VIIIa, VIIIb, IX, X, CrusI and CrusII). Secondary analyses included twenty-eight MRI phenotypes - sub-regional volumes of the cerebellum including the GM of the vermis and left and right lobules of each cerebellar region. L, left hemisphere; R, right hemisphere

In this study, we aimed to investigate the association between ASD PRS and the volumetric MRI phenotypes in the cerebellum and brainstem among adults at the population level. In addition to cerebellar and brainstem GM volume, we exploited the whole-brain MRI volumetric data to investigate the global volumes of the brain, cerebrospinal fluid (CSF), GM, and white matter (WM). Forty-four brain MRI phenotypes among ~ 31,000 participants from the UK Biobank (UKB) cohort were explored. First, we investigated sixteen **Total Volumes**, including total volumes of the *Brain*,* CSF*,* GM*,* WM*, GM of the whole *Cerebellum*,* Brainstem*, and ten regions of the cerebellum (*I_IV*,* V*,* VI*,* VIIb*,* VIIIa*,* VIIIb*,* IX*,* X*,* CrusI and CrusII*). Second, we investigated twenty-eight **Sub-regional Volumes** of the cerebellum including the GM of the *vermis* and *Left and Right lobules of each cerebellar region* (Fig. 1). We used the largest available GWAS dataset for ASD (*N* = 18,381 cases and *N* = 27,969 controls) [16] to construct ASD PRS for each subject. The utilization of increased GWAS sample size for PRS estimation, coupled with the large UKB sample of MRI measurements, robustly enhanced statistical power for examining the association between volumetric MRI phenotypes and ASD PRS within the European population.

## Methods and materials

### Participants

We employed baseline data obtained from the UKB project, one of the largest prospective population-based cohorts in Europe of > 500,000 subjects aged between 40 and 69 years recruited in 2006–2010 [36]. Our analyses focused exclusively on participants of European ancestry, as determined by self-report and multidimensional scaling (Data-field 22006), aligning with the ancestry criteria of the ASD GWAS conducted on individuals of European descent [37]. Exclusion criteria included withdrawal of consent, non-caucasian origin, failure to meet standard quality control criteria [38], sex mismatch between reported and genetic data (with sex as a covariate to control its influence), and outliers in terms of heterozygosity and genetic relatedness (further details provided below). We excluded participants with incomplete MRI data. Participants were also excluded from the present analysis if they reported having received a diagnosis of dementia, parkinson’s disease, multiple sclerosis, epilepsy, stroke, head or neurological injury, trauma, or other chronic neurological problems (nervous system infection, brain abscess, encephalitis, demyelinating disease, cerebral aneurysm, cerebral palsy, brain haemorrhage, or brain cancer) [39, 40]. Finally, our study model considered ~ 31,000 participants. Detailed participant enlisting illustrated in Supplementary Fig. 1. Ethical approval for UKB data collection was granted by the North-West Multicentre Research Ethics Committee (11/NW/0382), and the use of the data (under approved UKB application 30172) at our department was further approved by the Regional Ethics Committee of Uppsala, Sweden. Informed consents were obtained from all participants included in the study (https://biobank.ctsu.ox.ac.uk/crystal/field.cgi?id=200).

### Image acquisition and pre-processing

The present study made use of imaging-derived phenotypes generated by an image-processing pipeline developed and run on behalf of the UKB [41]. The T1-weighted structural MRI acquisition was performed with a 3T Siemens Skyra scanner (software platform VD13) equipped with a standard Siemens 32-channel RF receive head coil [42]. Global volumetric MRI phenotypes were obtained using the FAST segmentation tool [43]. Regional brain volumes were further extracted using the Harvard-Oxford cortical and subcortical atlases (https://fsl.fmrib.ox.ac.uk/fsl/fslwiki/Atlases), as well as the Diedrichsen cerebellar atlas (http://www.diedrichsenlab.org/imaging/propatlas.htm). All volumes are reported in mm^3^. Detailed information on MRI data acquisition and processing is available at: https://biobank.ctsu.ox.ac.uk/crystal/crystal/docs/brain_mri.pdf.

### Genotyping and genetic quality control

We utilized imputed genotyping data sourced from the UKB [44] in the study. Quality control (QC) procedures adhered to recommended protocols for PRS construction using UKB data [44, 45]. The analyses were restricted to SNPs meeting certain criteria: a minor allele frequency (MAF) > 0.5, an imputation information score (INFO) > 0.8, and genotype missingness < 0.02. Participants meeting genetic data QC standards within the UKB (Data-field 22020) [45] were included in the dataset. This involved excluding individuals with missing rates > 0.02 on autosomes, displaying sex discrepancies, heterozygosity outliers, genetic relatedness up to third-degree relatives, or individuals not identified as ‘White British’ based on genetic grouping (Data-field 22006) [45].

### Polygenic score construction

PRS computation were conducted for each participant in the UKB using the clumping and thresholding algorithms implemented in PRSice-2 (version 2.3.5) [46, 47]. The latest iPSYCH-Psychiatric Genomic Consortium (PGC) ASD GWAS summary statistics (November 2017), comprising 18,381 individuals with ASD and 27,969 individuals from the general population of European ancestry [16] was used as the base dataset for PRS construction. Following current guideline [48], we clumped SNPs in linkage disequilibrium (LD) using an LD-based *r*^*2*^ ≥ 0.1 and a 250 kb genomic distance. PRS for seven *P*_SNP_-value thresholds (*P* = 1, 0.75, 0.5, 0.25, 0.1, 0.01, and 0.001) were generated, and selected to achieve a balanced signal-to-noise ratio due to the highly polygenic nature of ASD [16]. The number of SNPs at each threshold is enlisted in Supplementary Tables 1, and a visual representation of these thresholds is provided in Supplementary Fig. 2.

### Statistical analyses of PRS associations with MRI phenotypes

We conducted linear regression analyses using standardized PRS as the independent variable (Eq. 1). The forty-four MRI phenotypes of interest were sorted into two separate analyses (Primary and Secondary) to explore structurally from overall to deep and, facilitate elaborative discussion of the findings. In the primary analyses, MRI phenotypes of interest were sixteen **Total Volumes**, including total volumes of the *Brain*,* CSF*,* GM*,* WM*, grey matter of the whole *Cerebellum*,* Brainstem*, and ten regions of the cerebellum (*I_IV*,* V*,* VI*,* VIIb*,* VIIIa*,* VIIIb*,* IX*,* X*,* CrusI and CrusII*). In the secondary analyses, MRI phenotypes of interest were twenty-eight **Sub-regional Volumes** of the cerebellum including the grey matter of the *left and right lobules of each cerebellar region (I_IV*,* V*,* VI*,* VIIb*,* VIIIa*,* VIIIb*,* IX*,* X*,* CrusI and CrusII)* and eight *vermis areas (VI*,* VIIb*,* VIIIa*,* VIIIb*,* IX*,* X*,* CrusI and CrusII)*. Covariates included age, age^2^, sex, genotyping batch, and the first 15 genetic principal components (Data-Field 22009). The age is the one recorded at the time of participant recruitment in 2006–2009, alongside the baseline genotyping data. The imaging data were obtained from the imaging visits from year 2014 and onwards [44]. Additionally, imaging sites/scanners (Data-Field 54) to control for multi-site scanner subtle effect [49] and, the x, y, and z coordinates of the head position in the scanner (Data-Field 25756–25758) to control for static-field heterogeneity were included [50]. Total volumes of the Brain, GM, WM, and CSF were analysed and normalised for head size (mm^3^). For the brainstem and all cerebellum phenotype regression models, intracranial volume (a composite of total volumes of Brain, GM, WM, and CSF) was considered as an additional covariate. All covariates were standardized. *P*-value significance (α) for the regression analyses (Eq. 1) was corrected for multiple testing including both, the number of regions (sixteen in primary analysis and twenty-eight in secondary analysis) and the number of *P*_SNP_-value thresholds using False-Discovery Rate (FDR) [51] at 5%. Statistical analyses were performed using R (version 3.6.3). MRI illustrations were made using MRIcroGL (version 1.2.20220720).

1 $$\eqalign{& MRI\,phenotypes \sim PRS\, + \,Age\, + \,Ag{e^2}\, + \,batch\, \cr & + \,15{\rm{ }}PCs\, + \,sites/scanners + \,X - coordinate\, + \,Y - coordinate \cr & + \,Z - coordinate \cr}$$

## Results

We investigated the association of ASD PRS and forty-four volumetric phenotypes including sixteen ‘Total Volumes’ and twenty-eight ‘Sub-regional Volumes’ on ~ 31,000 subjects (Supplementary Fig. 1). Participants included in the analysis comprised ~ 48% female and ~ 52% male, aged 40–70 years with a mean age of ~ 55 years.

### Total volumes

**Fig. 2:**
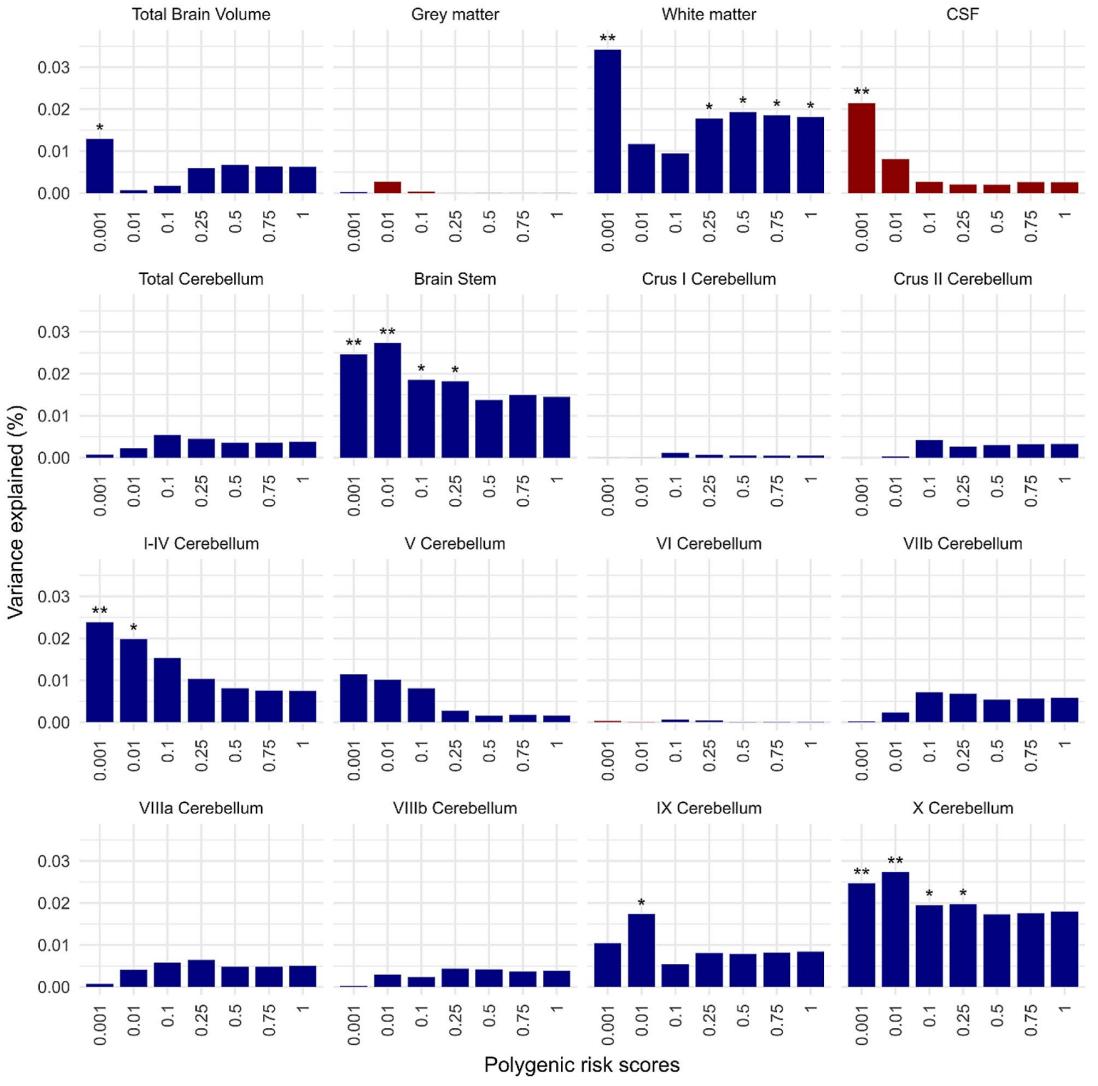
Associations between polygenic risk scores for ASD and the primary MRI phenotypes, *Total Volumes*. Bar charts of variance explained by ASD PRS (*R*^*2*^, y-axis) constructed at each of seven probability thresholds (0.001 ≥ *P*_SNP_ ≤1, x-axis) for each of the sixteen total volumes: total volumes of the Brain, CSF, GM, WM, grey matter of the whole Cerebellum, Brainstem and ten regions of cerebellum (I-IV, V, VI, VIIb, VIIIa, VIIIb, IX, X, CrusI and CrusII). Blue bars indicate negative associations and red bars positive associations; asterisks indicate *P* values for association after FDR correction: *P ≤ 0.05, **P ≤ 0.01, ***P ≤ 0.001. Polygenic risk scores for ASD were significantly negatively associated with the total volumes of the Brain, WM, Brainstem, and Cerebellar regions I-IV, IX, and X; and significantly positively associated with the CSF volume

**Fig. 3:**
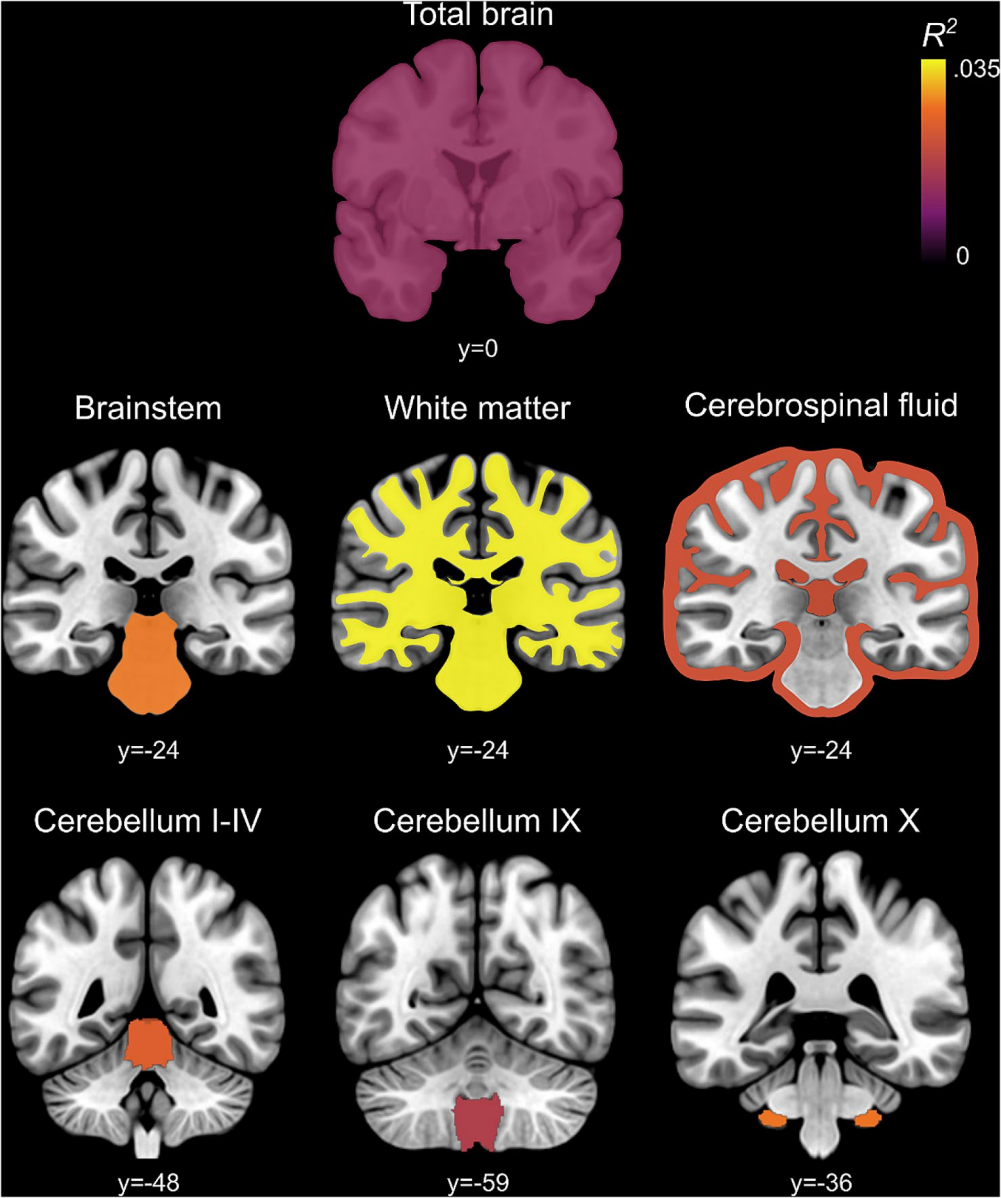
Anatomical localization of polygenic risk effects for ASD on the primary MRI phenotypes, *Total Volumes*. Coronal brain slices depicting the significant association between the total volumes and ASD PRS. The color bar indicates the highest variance (*R*^*2*^) explained by the ASD PRS for the primary MRI phenotypes with significant associations

Seven primary MRI phenotypes were significantly associated with the PRS for ASD at one or more *P*_SNP_ value thresholds (FDR = 5%) as illustrated in Fig. 2, and listed in Supplementary Table 2. Regions showing the highest variance explained by PRS are illustrated in Fig. 3. The proportion of variance explained by PRS varied between metrics in the range of 0.018% ≤ *R*^*2*^ ≤ 0.034%.

The total brain volumes were significantly negatively associated with the PRS constructed at *P*_SNP_ ≤0.001 explaining the highest variance (*β* = -861.46, *R*^*2*^ = 0.013%, 1,378 SNPs). Significant negative associations were also observed between the WM volumes and ASD PRS constructed at *P*_SNP_ ≤ 0.001, ≤ 0.25, ≤ 0.5, ≤ 0.75, and ≤ 1 (-606.91 ≤ *β* ≤ -776.65, *R*^*2*^ = 0.018–0.034%); highest variance explained with *P*_SNP_ ≤ 0.001 (1,378 SNPs, *R*^*2*^ = 0.034%). The inverse associations of brainstem volumes were found significant with the ASD PRS constructed at *P*_SNP_ ≤ 0.001, ≤ 0.01, ≤ 0.1 and ≤ 0.25 (-14.88 ≤ *β* ≤ -12.92, *R*^*2*^ = 0.018–0.027%); highest variance explained with *P*_SNP_ ≤ 0.01 (7,509 SNPs, *R*^*2*^ = 0.027%). Conversely, the CSF volumes were significantly positively associated with the PRS constructed with *P*_SNP_ ≤ 0.001 explaining the highest variance (*β* = 305.62, *R*^*2*^ = 0.022%, 1,378 SNPs).

The volumes of the cerebellar regions I-IV, IX, and X were significantly negatively associated with the PRS constructed at *P*_SNP_ ≤ 0.001 and ≤ 0.01 (-9.09 ≤ *β* ≤ -8.42), *P*_SNP_ ≤ 0.01 (*β****=*** -11.84), and *P*_SNP_ ≤ 0.001, ≤ 0.01, ≤ 0.1 and ≤ 0.25 (-2.85 ≤ *β* ≤ -2.54) respectively. The highest variance explained for the cerebellar regions I-IV, IX, and X were, respectively, with the PRS constructed at *P*_SNP_ ≤ 0.001 (*R*^*2*^ = 0.024%, 1,378 SNPs), *P*_SNP_ ≤ 0.01(*R*^*2*^ = 0.017%, 7,509 SNPs) and *P*_SNP_ ≤ 0.01 (*R*^*2*^ = 0.027%, 7,509 SNPs).

### Sub-regional volumes

**Fig. 4:**
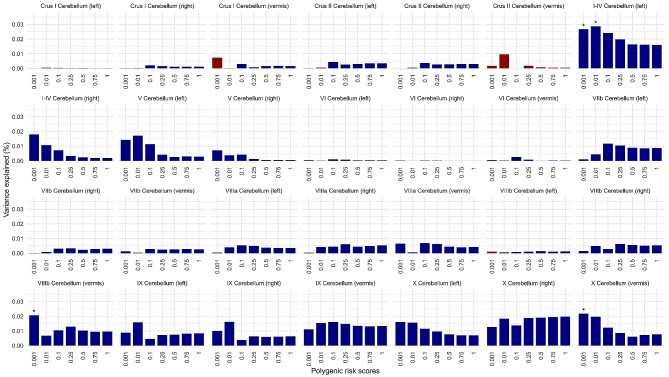
Associations between polygenic risk scores for ASD and the secondary MRI phenotypes, *Sub-regional Volumes*. Bar charts of variance explained by ASD PRS (*R*^*2*^, y-axis) constructed at each of seven probability thresholds (0.001 ≥ *P*_SNP_ ≤1, x-axis), for each of the twenty-eight cerebellar regional volumes: grey matter of the Left and Right lobules of each cerebellar region (I-IV, V, VI, VIIb, VIIIa, VIIIb, IX, X, CrusI and CrusII) and eight Vermis areas (VI, VIIb, VIIIa, VIIIb, IX, X, CrusI and CrusII). Blue bars indicate negative associations and red bars positive associations; asterisks indicate *P* values for association after FDR correction: *P ≤ 0.05, **P ≤ 0.01, ***P ≤ 0.001. Polygenic risk scores for ASD were significantly negatively associated with the volumes of the cerebellar regions I-IV (left), VIIIb (vermis) and X (vermis)

**Fig. 5:**
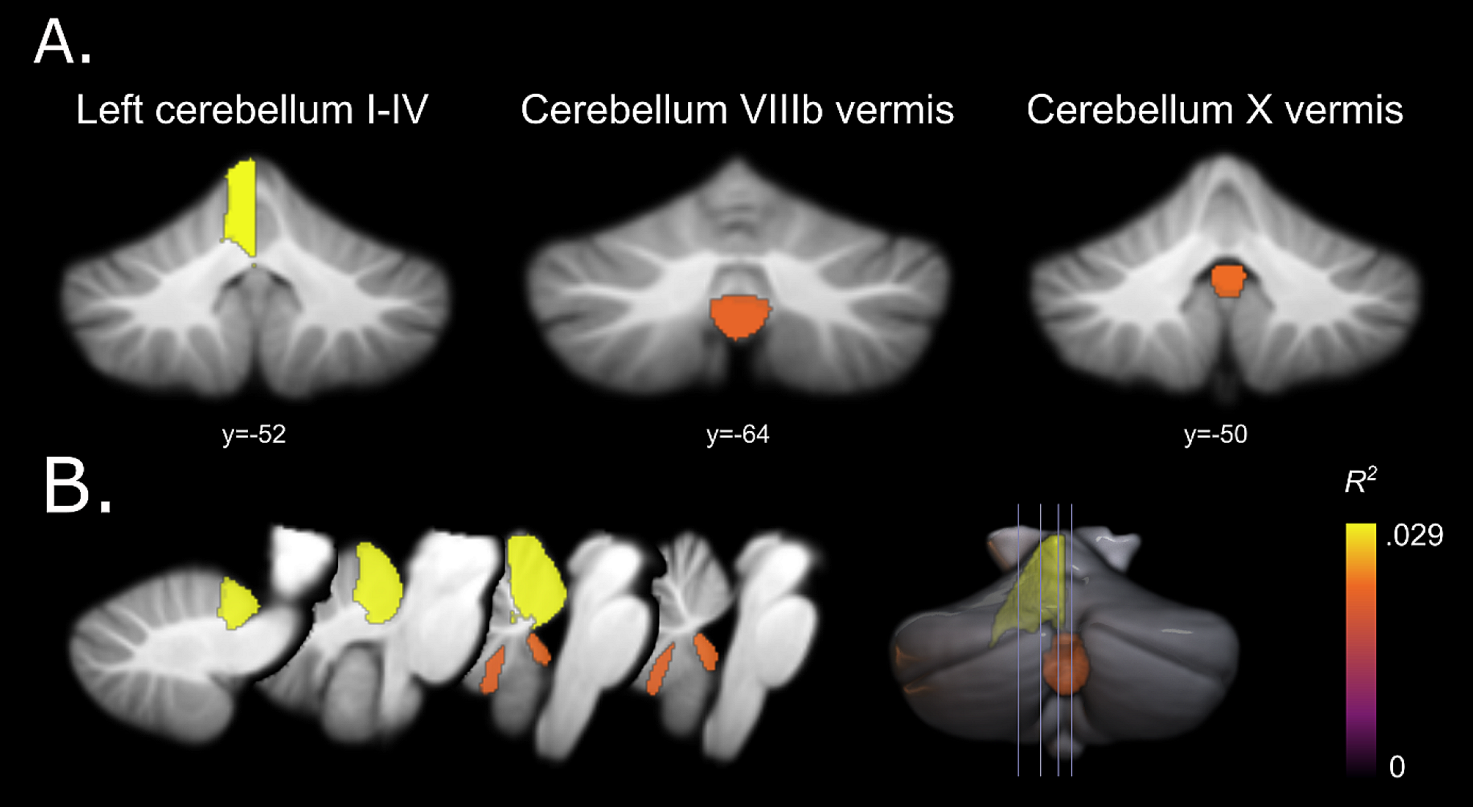
Anatomical localization of polygenic risk effects for ASD on the secondary MRI phenotypes, *Sub-regional Volumes*. (**A**) Coronal cerebellum sections, (**B**) sagittal sections and render view (right) depicting the significant association between the cerebellar sub-regional volumes and ASD PRS. The color bar indicates the highest variance (*R*^*2*^) explained by the ASD PRS for the secondary MRI phenotypes with significant associations

Three secondary MRI phenotypes, including the left cerebellar lobule I-IV, vermis VIIIb, and vermis X, were significantly associated with the PRS for ASD at one or more *P*_SNP_ value thresholds (FDR = 5%) (Fig. 4, Supplementary Table 3), Regions showing the highest variance explained by PRS are illustrated in Fig. 5.

The proportion of regional variance explained by PRS varied between metrics in the range 0.021% ≤ *R*^*2*^ ≤ 0.029% with − 5.07 ≤ β ≤ -0.66. The significant relation between the volumes of the left cerebellar lobule I-IV, vermis VIIIb, and vermis X was negative, with PRS constructed at *P*_SNP_ ≤ 0.001 and ≤ 0.01 (-5.07 ≤ *β* ≤ -4.82), *P*_SNP_ ≤ 0.001 (*β* = -1.25),and *P*_SNP_ ≤ 0.001 (*β* = -0.66) ,respectively; and the highest variance explained with the ASD PRS constructed at *P*_SNP_ ≤ 0.01 (*R*^*2*^ = 0.029%, 7,509 SNPs, ), *P*_SNP_ ≤ 0.001 (*R*^*2*^ = 0.021%, 1,378 SNPs), and *P*_SNP_ ≤ 0.001 (*R*^*2*^ = 0.022%, 1,378 SNPs), respectively.

### Sensitivity analysis

We repeated the association analyses between ASD PRS and all forty-four MRI volumetric phenotypes (Total volumes and Sub-regional volumes), excluding the individuals (*N* = 25) with self-reported physician-posed ASD diagnosis (Data-field 20544) in UKB. The results remained consistent and statistically significant as the main analyses (Supplementary Table 4).

## Discussion

This study is the first to investigate the linkage between the genetic propensity of ASD and forty-four volumetric brain MRI phenotypes, with a focus on dissecting the association between ASD PRS and volumetric shifts in the cerebellum and brainstem in adults. Additionally, we directly examined the correlation between the genetic predisposition for ASD and the global volumes of the brain, GM, WM, and CSF in an adult population. Beyond prior evidence on the neuroanatomical atypicalities of ASD, this study robustly highlights neuroimaging findings in relation to the genetic propensity of ASD, at the population level in an adult European cohort with substantial statistical power.

Our investigation using PRS constructed from the recently published GWAS for ASD indicate that adults genetically predisposed to ASD exhibit a significantly reduced total brain volume, WM volume, and brainstem volume, alongside with an increased CSF volume. Specifically, individuals with higher ASD PRS show a reduced volume in the cerebellar lobules I-IV, IX, and X, as well as vermes VIIIb and X. Our findings intriguingly highlight changes in the mature brain of adults at risk for ASD, while previous studies mostly investigated young developing brains. Most of these studies reported increased volumes among participants with ASD and are based on children or adolescents [2–4]. However, volumetric changes with age among individuals with ASD have been widely discussed [3, 4]. An accelerated growth leading to an enlarged brain in children with ASD has been observed during early life, while a decrease in brain volume is found over the developmental period in which typically developing children experience slight increases [29, 31]. There is also consensus that the underlying pathology of brain development in ASD involves increased growth of both GM and WM during early childhood, followed by a rapid synaptic pruning process during early adolescence [52, 53].

We show that adults with a higher genetic predisposition for ASD are more likely to have a reduced total brain volume. Volumetric analyses in adults with ASD reporting total brain volume changes are rare. To the best of our knowledge, our study is the first to report a total brain volume reduction in adults at risk for ASD. Additionally, we show that adults who are genetically predisposed for ASD show reduced WM volumes. There are mixed reports on total WM volume in ASD brains, indicating overall or region-specific increases that have been observed [4]. However, a significant reduction of the mean volume of cortical WM was reported in a longitudinal study comparing participants with ASD and typically developing controls, which showed a developmental curve reflecting reduction with age [33]. Our findings regarding a reduced total volume of brain and WM in adults genetically predisposed to ASD are in line with these findings. It can be hypothesized that individuals with ASD continue to show a higher reduction in brain volumes with age compared to individuals without ASD.

We found a substantial correlation between reduced brainstem volume and elevated ASD PRS. Our findings support the most commonly reported structural alteration of the brainstem in ASD, as described in a recent review on ASD neuroimaging studies [10]. The review summarized mixed results in relation to the brainstem size in ASD. However, a reduction of the total volume or of at least one of the brainstem components was most commonly observed [10]. The contribution of an abnormal brainstem neurotransmission to ASD symptoms becomes well evident when co-occurring features of ASD such as sensorimotor challenges or psychophysiological atypicalities are examined [9]. Moreover, the age effect in ASD brainstem volumetric change is highlighted in the mentioned review, as indicated by the difference in outcome between adult and child populations. Additionally, a reduction in this difference with an increase in age is observed in longitudinal studies [10].

Our findings on increased CSF volume are in line with previous reports on adult brain changes in ASD [54, 55]. A recent review paper concluded as well that CSF volume is increased in ASD [25], as hitherto mostly observed in children or adolescents. The consistent evidence of increased CSF volume in ASD [25, 54, 55], has not yet been thoroughly explored in relation to ASD pathophysiology. However, in a recent review Shen (2018) discussed the potential links between ASD and CSF abnormalities and concluded that CSF could be a potential early biomarker for ASD and its subtypes. Infants with ASD, compared to typical infants, exhibit factors such as an imbalance in CSF production and drainage, slower CSF recycling in early life, and a higher ratio of CSF volume in infancy. These collectively contribute to a reduced ability to eliminate inflammatory metabolites and toxins, eventually leading to increased levels of Aβ in neurons, sleep problems, and cognitive impairment involving the lymphatic system [56].

Our investigation on the cerebellum demonstrates that adults genetically predisposed to ASD anatomically present reduced cerebellar volume in regions including the lobules I-IV, specifically in the left hemisphere, IX, and X, as well as the vermes VIIIb and X. Previously, a decreased GM volume in the anterior lobe of the cerebellum (lobule I-V) and lobule IX has been reported in children with ASD [8]. Our findings robustly show a similar, reduced volume of regions I-IV and IX among adults with a genetic predisposition for ASD. While the lobule I-V is involved in increased stereotypical and repetitive behavior, and lobule IX is correlated with poorer communication skills as measured by standard ASD scales [8], we posit that the cerebellar differences observed in children remain at the adult age.

While we observed that a reduced GM of the cerebellar region X is significantly associated with a genetic propensity for ASD, hardly any studies investigated this region in adults with ASD. Recent study including 58 individuals with ASD, and 34 typically developing individuals aged 8–30 years reported a reduced volume in the cerebellar region X (right lobule), which was inversely associated with ASD symptom severity [57]. Furthermore, post-mortem studies also reported dysplasia and hypoplasia of the cerebellar region X, indicating cell migration abnormalities [58]. Lower Purkinje cell density in region X is also possibly involved in restricted eye movement behaviour in ASD [59]. Our findings on the association between the cerebellar region X volume and ASD PRS support these findings.

While investigating the cerebellar volumes at the sub-regional level, we observed that adults with a genetic propensity for ASD have a reduced volume in the left cerebellar region I-IV, and vermes VIIIb and X. There are very few studies on adult ASD investigating the sub-regional level of the cerebellum. Overall, the cerebellar vermis volume has been consistently observed to be reduced in ASD [25]. However, our investigation of the cerebellar vermis region among adults suggests that areas VIIIb, and X are significantly associated with ASD PRS. Previously, an increased volume of vermis VIIIb among children with ASD was observed [8]. However, we found a decreased volume among adults. Thus, our results support a previous report that showed a decreased GM in vermis VIIIb occurring in early childhood and no effect with increasing age [60]. Reduced cerebellar vermis volume is associated with ASD symptomatology, including impaired global development, cognition, expressive language, and gross and fine motor skills, as well as behavior problems [61].

We also observed the GM volume of the left I-IV cerebellar region to be significantly negatively associated with the ASD PRS. Interestingly, reduced GM in the right I-IV cerebellar region has been observed to correlate with higher severity of results in ASD diagnostic interviews and social interaction scores in children with ASD [8]. Altered lateralized neurodevelopment of brain has been reported in ASD [62], which explains the observed left-right asymmetry. Further longitudinal neuroimaging studies are required to explain the age effect on variations in hemispheric patterns of volumetric changes. While we observed significant associations between ASD PRS and the left cerebellar regions I-IV, as well as vermes VIIIb and X, the total volume of the cerebellum was not significantly associated with ASD PRS. A plausible interpretation of such finding is that genetic factors implicated in ASD may specifically affect the development or size of certain cerebellar regions rather than the entire cerebellum uniformly. It is intriguing to speculate that these regions are particularly sensitive to the genetic variants associated with ASD.

Our findings robustly infer the understanding that age has a significant effect on the developmental trajectory of ASD [3, 4] and support the theory of age-specific anatomic abnormalities in ASD [29, 31]. Our study suggest the age-specific deceleration to be predominant in adult life in ASD, resulting in reduced volume of the brain on the overall and specifically on the cerebellar regional level. Our findings of volume reduction on the cerebellar sub-regional level robustly support the previous consistent findings in post-mortem analyses of ASD individuals, reporting reduced Purkinje cell size and number regardless of age, sex, or cognitive ability [5].

Strengths of this study include the application of a powerful PRS approach to investigate the association between genetic predisposition for ASD and brain structure in the adult population. We utilized the recent-largest GWAS for ASD including multiple *P*_SNP_-value thresholds for the inclusion of risk variants in the PRS construction. One of the most methodologically diverse and largest MRI datasets to date was used to investigate the PRS associations with brain structures. The findings of the present study should, however, be interpreted with caution, as factors like an insult or socio-emotional behavioural damage during life [63], IQ and behavioral phenotypes [64] of participants have not been taken into account in the study, which may have contributed to the regional adult cerebellar volumetric differences. A limitation of the study is that the ASD PRS only explains a relatively small variance of 2.5% compared to a SNP-based heritability of 11% in ASD [16]. Advancements in future GWAS with even larger sample size may potentiate our understanding of the associations observed in this study. This must also be acknowledged that, the findings in the study are based on the genetic predisposition for ASD and not based on ASD diagnoses. While our findings are in line with and support previous findings, future studies in similar direction on the ground of diagnosed ASD adult cases may provide more emphasized and elaborated understanding. Furthermore, the UK Biobank represents an aging cohort predominantly of European ancestry, which is characterized by higher average socioeconomic status and better health compared to the broader population [65]. Thus, the generalizability of the presented findings may need further explorations in more diverse populations.

In summary, this was the first study to investigate associations between brain volumes focusing on the cerebellum and brainstem, and polygenic risk scores for ASD in the adult population with high statistical power, utilizing both genetic and imaging data. We show that certain global volumes such as the whole brain, WM, brainstem, CSF, and volumes in cerebellar lobules I-IV, IX, and X, as well as vermes VIIIb, and X are significantly predicted by ASD polygenic risk scores. These findings show that the cumulative genetic load for ASD is associated with considerable changes in the adult brain. ASD typically manifests early in life and entails significant neurodevelopmental symptoms, often accompanied by structural changes in the brain. This supports a possible explanation for our observations, suggesting that genetic susceptibility to ASD may lead to notable structural variations in the brain, even among individuals who do not exhibit the full spectrum of the disorder. The findings may robustly be confirmed by future translational MRI and histological studies involving human post-mortem data or animal models of genetic susceptibility for ASD.

## Electronic supplementary material

Below is the link to the electronic supplementary material.

Supplementary Material

## Data Availability

All data supporting this study is available from the UK Biobank (https://www.ukbiobank.ac.uk/) upon request. All data generated during this study are included in this paper.

## References

[1] Diagnostic and statistical manual of mental disorders: DSM-5™, 5th ed (pp. xliv, 947). (2013). American Psychiatric Publishing, Inc. 10.1176/appi.books.9780890425596.

[2] Chen R, Jiao Y, Herskovits EH. Structural MRI in Autism Spectrum Disorder. Pediatr Res. 2011;69(8):63–8. 10.1203/PDR.0b013e318212c2b3. 10.1203/PDR.0b013e318212c2b3 PMC3081653 21289538

[3] Faizo NL. A narrative review of MRI changes correlated to signs and symptoms of autism. Medicine. 2022;101(34):e30059. 10.1097/MD.0000000000030059. 36042586 10.1097/MD.0000000000030059 PMC9410622

[4] Rafiee F, Rezvani Habibabadi R, Motaghi M, Yousem DM, Yousem IJ. Brain MRI in Autism Spectrum Disorder: Narrative Review and recent advances. J Magn Reson Imaging. 2022;55(6):1613–24. 10.1002/jmri.27949. 34626442 10.1002/jmri.27949

[5] Fatemi SH, Aldinger KA, Ashwood P, Bauman ML, Blaha CD, Blatt GJ, Chauhan A, Chauhan V, Dager SR, Dickson PE, Estes AM, Goldowitz D, Heck DH, Kemper TL, King BH, Martin LA, Millen KJ, Mittleman G, Mosconi MW, … Welsh JP. Consensus paper: Pathological role of the cerebellum in autism. The Cerebellum. 2012;11(3):777–807. 10.1007/s12311-012-0355-9. 10.1007/s12311-012-0355-9 PMC3677555 22370873

[6] Wang SS-H, Kloth AD, Badura A. The Cerebellum, sensitive periods, and Autism. Neuron. 2014;83(3):518–32. 10.1016/j.neuron.2014.07.016. 25102558 10.1016/j.neuron.2014.07.016 PMC4135479

[7] Willsey AJ, Sanders SJ, Li M, Dong S, Tebbenkamp AT, Muhle RA, Reilly SK, Lin L, Fertuzinhos S, Miller JA, Murtha MT, Bichsel C, Niu W, Cotney J, Ercan-Sencicek AG, Gockley J, Gupta AR, Han W, He X, … State MW. (2013). Coexpression networks implicate human midfetal deep cortical projection neurons in the pathogenesis of autism. Cell 155(5): 997–1007. 10.1016/j.cell.2013.10.020. 10.1016/j.cell.2013.10.020 PMC3995413 24267886

[8] D’Mello AM, Crocetti D, Mostofsky SH, Stoodley CJ. Cerebellar gray matter and lobular volumes correlate with core autism symptoms. NeuroImage: Clin. 2015;7:631–9. 10.1016/j.nicl.2015.02.007. 25844317 10.1016/j.nicl.2015.02.007 PMC4375648

[9] Dadalko OI, Travers BG. Evidence for Brainstem contributions to Autism Spectrum disorders. Front Integr Nuerosci. 2018;12. 10.3389/fnint.2018.00047. 10.3389/fnint.2018.00047 PMC6180283 30337860

[10] Seif A, Shea C, Schmid S, Stevenson R. A. A systematic review of Brainstem contributions to Autism Spectrum Disorder. Front Integr Nuerosci. 2021;15. 10.3389/fnint.2021.760116. 10.3389/fnint.2021.760116 PMC8591260 34790102

[11] Genovese A, Butler MG. The Autism Spectrum: behavioral, Psychiatric and Genetic associations. Genes. 2023;14(3). 10.3390/genes14030677. 10.3390/genes14030677 PMC10048473 36980949

[12] Hashem S, Nisar S, Bhat AA, Yadav SK, Azeem MW, Bagga P, Fakhro K, Reddy R, Frenneaux MP, Haris M. Genetics of structural and functional brain changes in autism spectrum disorder. Translational Psychiatry. 2020;10(1):1–17. 10.1038/s41398-020-00921-3. 32661244 10.1038/s41398-020-00921-3 PMC7359361

[13] Pretzsch CM, Ecker C. Structural neuroimaging phenotypes and associated molecular and genomic underpinnings in autism: a review. Front NeuroSci. 2023;17. 10.3389/fnins.2023.1172779. 10.3389/fnins.2023.1172779 PMC10347684 37457001

[14] Nisar S, Haris M. Neuroimaging genetics approaches to identify new biomarkers for the early diagnosis of autism spectrum disorder. Mol Psychiatry. 2023;1–14. 10.1038/s41380-023-02060-9. 10.1038/s41380-023-02060-9 PMC11041805 37069342

[15] Kainer D, Templeton AR, Prates ET, Jacboson D, Allan ERO, Climer S, Garvin MR. Structural variants identified using non-mendelian inheritance patterns advance the mechanistic understanding of autism spectrum disorder. Hum Genet Genomics Adv. 2023;4(1):100150. 10.1016/j.xhgg.2022.100150. 10.1016/j.xhgg.2022.100150 PMC9634371 36340933

[16] Grove J, Ripke S, Als TD, Mattheisen M, Walters RK, Won H, Pallesen J, Agerbo E, Andreassen OA, Anney R, Awashti S, Belliveau R, Bettella F, Buxbaum JD, Bybjerg-Grauholm J, Bækvad-Hansen M, Cerrato F, Chambert K, Christensen JH, … Børglum AD. (2019). Identification of common genetic risk variants for autism spectrum disorder. Nature Genetics 51(3): 431–444. 10.1038/s41588-019-0344-8. 10.1038/s41588-019-0344-8 PMC6454898 30804558

[17] Martin AR, Daly MJ, Robinson EB, Hyman SE, Neale BM. Predicting Polygenic Risk of Psychiatric disorders. Mol Mech Affect Disturb. 2019;86(2):97–109. 10.1016/j.biopsych.2018.12.015. 10.1016/j.biopsych.2018.12.015 PMC6599546 30737014

[18] Khundrakpam B, Vainik U, Gong J, Al-Sharif N, Bhutani N, Kiar G, Zeighami Y, Kirschner M, Luo C, Dagher A, Evans A. Neural correlates of polygenic risk score for autism spectrum disorders in general population. Brain Commun. 2020;2(2):fcaa092. 10.1093/braincomms/fcaa092. 32954337 10.1093/braincomms/fcaa092 PMC7475696

[19] Alemany S, Blok E, Jansen PR, Muetzel RL, White T. Brain morphology, autistic traits, and polygenic risk for autism: a population-based neuroimaging study. Autism Res. 2021;14(10):2085–99. 10.1002/aur.2576. 34309210 10.1002/aur.2576

[20] Ranlund S, Rosa MJ, de Jong S, Cole JH, Kyriakopoulos M, Fu CHY, Mehta MA, Dima D. Associations between polygenic risk scores for four psychiatric illnesses and brain structure using multivariate pattern recognition. NeuroImage: Clin. 2018;20:1026–36. 10.1016/j.nicl.2018.10.008. 30340201 10.1016/j.nicl.2018.10.008 PMC6197704

[21] Traut N, Beggiato A, Bourgeron T, Delorme R, Rondi-Reig L, Paradis A-L, Toro R. Cerebellar volume in Autism: literature Meta-analysis and analysis of the Autism Brain Imaging Data Exchange Cohort. Social Behav Autism. 2018;83(7):579–88. 10.1016/j.biopsych.2017.09.029. 10.1016/j.biopsych.2017.09.029 29146048

[22] Durkut M, Blok E, Suleri A, White T. The longitudinal bidirectional relationship between autistic traits and brain morphology from childhood to adolescence: a population-based cohort study. Mol Autism. 2022;13(1):31. 10.1186/s13229-022-00504-7. 35790991 10.1186/s13229-022-00504-7 PMC9258195

[23] Liu J, Yao L, Zhang W, Xiao Y, Liu L, Gao X, Shah C, Li S, Tao B, Gong Q, Lui S. Gray Matter abnormalities in pediatric autism spectrum disorder: a meta-analysis with signed differential mapping. European Child Adolescent Psychiatry. 2017;26(8):933–45. 10.1007/s00787-017-0964-4. 28233073 10.1007/s00787-017-0964-4

[24] Yang Q, Huang P, Li C, Fang P, Zhao N, Nan J, Wang B, Gao W, Cui L-B. Mapping alterations of gray matter volume and white matter integrity in children with autism spectrum disorder: evidence from fMRI findings. NeuroReport. 2018;29(14):1188–92. 10.1097/WNR.0000000000001094. 30001226 10.1097/WNR.0000000000001094

[25] Pagnozzi AM, Conti E, Calderoni S, Fripp J, Rose SE. A systematic review of structural MRI biomarkers in autism spectrum disorder: a machine learning perspective. Int J Dev Neurosci. 2018;71(1):68–82. 10.1016/j.ijdevneu.2018.08.010. 30172895 10.1016/j.ijdevneu.2018.08.010

[26] Webb SJ, Sparks B-F, Friedman SD, Shaw DWW, Giedd J, Dawson G, Dager SR. Cerebellar vermal volumes and behavioral correlates in children with autism spectrum disorder. Psychiatry Res: Neuroimaging. 2009;172(1):61–7. 10.1016/j.pscychresns.2008.06.001. 10.1016/j.pscychresns.2008.06.001 PMC2676721 19243924

[27] Laidi C, Floris DL, Tillmann J, Elandaloussi Y, Zabihi M, Charman T, Wolfers T, Durston S, Moessnang C, Dell’Acqua F, Ecker C, Loth E, Murphy D, Baron-Cohen S, Buitelaar JK, Marquand AF, Beckmann CF, Frouin V, Leboyer M, … Simonoff E. Cerebellar atypicalities in autism? Brain Development and Communication in Autism Spectrum Disorder 2022;92 (8): 674–682. 10.1016/j.biopsych.2022.05.020. 10.1016/j.biopsych.2022.05.020 36137706

[28] Fernandez L, Burmester A, Duque JD, Silk TJ, Hyde CE, Kirkovski M, Enticott PG, Caeyenberghs K. Examination of cerebellar Grey-Matter volume in children with neurodevelopmental disorders: a coordinated analysis using the ACAPULCO Algorithm. Cerebellum. 2023;22(6):1243–9. 10.1007/s12311-022-01503-3. 36482028 10.1007/s12311-022-01503-3

[29] Courchesne E, Campbell K, Solso S. Brain growth across the life span in autism: age-specific changes in anatomical pathology. Brain Res. 2011;1380:138–45. 10.1016/j.brainres.2010.09.101. 20920490 10.1016/j.brainres.2010.09.101 PMC4500507

[30] Duerden EG, Mak-Fan KM, Taylor MJ, Roberts SW. Regional differences in grey and white matter in children and adults with autism spectrum disorders: an activation likelihood estimate (ALE) meta-analysis. Autism Res. 2012;5(1):49–66. 10.1002/aur.235. 22139976 10.1002/aur.235

[31] Ecker C, Bookheimer SY, Murphy DGM. Neuroimaging in autism spectrum disorder: brain structure and function across the lifespan. Lancet Neurol. 2015;14(11):1121–34. 10.1016/S1474-4422(15)00050-2. 25891007 10.1016/S1474-4422(15)00050-2

[32] Yang X, Si T, Gong Q, Qiu L, Jia Z, Zhou M, Zhao Y, Hu X, Wu M, Zhu H. Brain gray matter alterations and associated demographic profiles in adults with autism spectrum disorder: a meta-analysis of voxel-based morphometry studies. Australian New Z J Psychiatry. 2016;50(8):741–53. 10.1177/0004867415623858. 26769980 10.1177/0004867415623858

[33] Lange N, Travers BG, Bigler ED, Prigge MBD, Froehlich AL, Nielsen JA, Cariello AN, Zielinski BA, Anderson JS, Fletcher PT, Alexander AA, Lainhart JE. Longitudinal volumetric brain changes in Autism Spectrum Disorder ages 6–35 years. Autism Res. 2015;8(1):82–93. 10.1002/aur.1427. 25381736 10.1002/aur.1427 PMC4344386

[34] Robinson EB, Koenen KC, McCormick MC, Munir K, Hallett V, Happé F, Plomin R, Ronald A. Evidence that autistic traits show the same etiology in the General Population and at the quantitative extremes (5%, 2.5%, and 1%). Arch Gen Psychiatry. 2011;68(11):1113–21. 10.1001/archgenpsychiatry.2011.119. 22065527 10.1001/archgenpsychiatry.2011.119 PMC3708488

[35] Robinson EB, St Pourcain B, Anttila V, Kosmicki JA, Bulik-Sullivan B, Grove J, Maller J, Samocha KE, Sanders SJ, Ripke S, Martin J, Hollegaard MV, Werge T, Hougaard DM, Neale BM, Evans DM, Skuse D, Mortensen PB, Børglum AD, … Daly MJ. Genetic risk for autism spectrum disorders and neuropsychiatric variation in the general population. Nat Genet 2016;48(5):552–555. 10.1038/ng.3529. 10.1038/ng.3529 PMC4986048 26998691

[36] Sudlow C, Gallacher J, Allen N, Beral V, Burton P, Danesh J, Downey P, Elliott P, Green J, Landray M, Liu B, Matthews P, Ong G, Pell J, Silman A, Young A, Sprosen T, Peakman T, Collins R. UK Biobank: an Open Access Resource for identifying the causes of a wide range of Complex diseases of Middle and Old Age. PLoS Med. 2015;12(3):e1001779. 10.1371/journal.pmed.1001779. 25826379 10.1371/journal.pmed.1001779 PMC4380465

[37] Martin AR, Kanai M, Kamatani Y, Okada Y, Neale BM, Daly MJ. Clinical use of current polygenic risk scores may exacerbate health disparities. Nat Genet. 2019;51(4):584–91. 10.1038/s41588-019-0379-x. 30926966 10.1038/s41588-019-0379-x PMC6563838

[38] Marees AT, de Kluiver H, Stringer S, Vorspan F, Curis E, Marie-Claire C, Derks EM. A tutorial on conducting genome-wide association studies: quality control and statistical analysis. Int J Methods Psychiatr Res. 2018;27(2):e1608. 10.1002/mpr.1608. 29484742 10.1002/mpr.1608 PMC6001694

[39] Shang X, Zhang X, Huang Y, Zhu Z, Zhang X, Liu J, Wang W, Tang S, Yu H, Ge Z, Yang X, He M. Association of a wide range of individual chronic diseases and their multimorbidity with brain volumes in the UK Biobank: a cross-sectional study. eClinicalMedicine. 2022;47:101413. 10.1016/j.eclinm.2022.101413. 35518119 10.1016/j.eclinm.2022.101413 PMC9065617

[40] Cox SR, Lyall DM, Ritchie SJ, Bastin ME, Harris MA, Buchanan CR, Fawns-Ritchie C, Barbu MC, de Nooij L, Reus LM, Alloza C, Shen X, Neilson E, Alderson HL, Hunter S, Liewald DC, Whalley HC, McIntosh AM, Lawrie SM, … Deary IJ. Associations between vascular risk factors and brain MRI indices in UK Biobank. Eur Heart J. 2019;40(28):2290–2300. 10.1093/eurheartj/ehz100. 10.1093/eurheartj/ehz100 PMC6642726 30854560

[41] Alfaro-Almagro F, Jenkinson M, Bangerter NK, Andersson JLR, Griffanti L, Douaud G, Sotiropoulos SN, Jbabdi S, Hernandez-Fernandez M, Vallee E, Vidaurre D, Webster M, McCarthy P, Rorden C, Daducci A, Alexander DC, Zhang H, Dragonu I, Matthews PM, … Smith SM. Image processing and Quality Control for the first 10,000 brain imaging datasets from UK Biobank. NeuroImage 2018;166:400–424. 10.1016/j.neuroimage.2017.10.034. 10.1016/j.neuroimage.2017.10.034 PMC5770339 29079522

[42] Miller KL, Alfaro-Almagro F, Bangerter NK, Thomas DL, Yacoub E, Xu J, Bartsch AJ, Jbabdi S, Sotiropoulos SN, Andersson JLR, Griffanti L, Douaud G, Okell TW, Weale P, Dragonu I, Garratt S, Hudson S, Collins R, Jenkinson M, … Smith SM. Multimodal population brain imaging in the UK Biobank prospective epidemiological study. Nat Neurosci. 2016;19(11):1523–1536. 10.1038/nn.4393. 10.1038/nn.4393 PMC5086094 27643430

[43] Zhang Y, Brady M, Smith S. Segmentation of brain MR images through a hidden Markov random field model and the expectation-maximization algorithm. IEEE Trans Med Imag. 2001;20(1):45–57. 10.1109/42.906424. 10.1109/42.906424 11293691

[44] Bycroft C, Freeman C, Petkova D, Band G, Elliott LT, Sharp K, Motyer A, Vukcevic D, Delaneau O, O’Connell J, Cortes A, Welsh S, Young A, Effingham M, McVean G, Leslie S, Allen N, Donnelly P, Marchini J. The UK Biobank resource with deep phenotyping and genomic data. Nature. 2018;562(7726):203–9. 10.1038/s41586-018-0579-z. 30305743 10.1038/s41586-018-0579-z PMC6786975

[45] Collister JA, Liu X, Clifton L. Calculating polygenic risk scores (PRS) in UK Biobank: a practical guide for epidemiologists. Front Genet. 2022;13. 10.3389/fgene.2022.818574. 10.3389/fgene.2022.818574 PMC8894758 35251129

[46] Choi SW, O’Reilly PF. PRSice-2: polygenic risk score software for biobank-scale data. GigaScience. 2019;8(7):giz082. 10.1093/gigascience/giz082. 31307061 10.1093/gigascience/giz082 PMC6629542

[47] Euesden J, Lewis CM, O’Reilly PF. PRSice: polygenic risk score software. Bioinformatics. 2015;31(9):1466–8. 10.1093/bioinformatics/btu848. 25550326 10.1093/bioinformatics/btu848 PMC4410663

[48] Wray NR, Lee SH, Mehta D, Vinkhuyzen AAE, Dudbridge F, Middeldorp CM. Research Review: polygenic methods and their application to psychiatric traits. J Child Psychol Psychiatry. 2014;55(10):1068–87. 10.1111/jcpp.12295. 25132410 10.1111/jcpp.12295

[49] Alfaro-Almagro F, McCarthy P, Afyouni S, Andersson JLR, Bastiani M, Miller KL, Nichols TE, Smith SM. Confound modelling in UK Biobank brain imaging. NeuroImage. 2021;224:117002. 10.1016/j.neuroimage.2020.117002. 32502668 10.1016/j.neuroimage.2020.117002 PMC7610719

[50] Smith SM, Nichols TE. Statistical challenges in Big Data Human Neuroimaging. Neuron. 2018;97(2):263–8. 10.1016/j.neuron.2017.12.018. 29346749 10.1016/j.neuron.2017.12.018

[51] Benjamini Y, Hochberg Y. Controlling the false Discovery rate: a practical and powerful Approach to multiple testing. J Roy Stat Soc: Ser B (Methodol). 1995;57(1):289–300. 10.1111/j.2517-6161.1995.tb02031.x.

[52] Kim H-J, Cho M-H, Shim WH, Kim JK, Jeon E-Y, Kim D-H, Yoon S-Y. Deficient autophagy in microglia impairs synaptic pruning and causes social behavioral defects. Mol Psychiatry. 2017;22(11):1576–84. 10.1038/mp.2016.103. 27400854 10.1038/mp.2016.103 PMC5658669

[53] Ziats MN, Edmonson C, Rennert OM. (2015). The autistic brain in the context of normal neurodevelopment. Front Neuroanat. 9. 10.3389/fnana.2015.00115. 10.3389/fnana.2015.00115 PMC4548149 26379512

[54] Hallahan B, Daly EM, McAlonan G, Loth E, Toal F, O’Brien F, Robertson D, Hales S, Murphy C, Murphy KC, Murphy DGM. Brain morphometry volume in autistic spectrum disorder: a magnetic resonance imaging study of adults. Psychol Med. 2009;39(2):337–46. 10.1017/S0033291708003383. 18775096 10.1017/S0033291708003383

[55] Katuwal GJ, Baum SA, Cahill ND, Dougherty CC, Evans E, Evans DW, Moore GJ, Michael AM. Inter-method discrepancies in brain volume estimation may drive inconsistent findings in Autism. Front NeuroSci. 2016;10. 10.3389/fnins.2016.00439. 10.3389/fnins.2016.00439 PMC5043189 27746713

[56] Shen MD. Cerebrospinal fluid and the early brain development of autism. J Neurodevelopmental Disorders. 2018;10(1):39. 10.1186/s11689-018-9256-7. 10.1186/s11689-018-9256-7 PMC6292033 30541429

[57] McKinney WS, Kelly SE, Unruh KE, Shafer RL, Sweeney JA, Styner M, Mosconi MW. Cerebellar volumes and Sensorimotor Behavior in Autism Spectrum Disorder. Front Integr Nuerosci. 2022;16. 10.3389/fnint.2022.821109. 10.3389/fnint.2022.821109 PMC9113114 35592866

[58] Wegiel J, Kuchna I, Nowicki K, Imaki H, Wegiel J, Marchi E, Ma SY, Chauhan A, Chauhan V, Bobrowicz TW, de Leon M, Louis LAS, Cohen IL, London E, Brown WT, Wisniewski T. The neuropathology of autism: defects of neurogenesis and neuronal migration, and dysplastic changes. Acta Neuropathol. 2010;119(6):755–70. 10.1007/s00401-010-0655-4. 20198484 10.1007/s00401-010-0655-4 PMC2869041

[59] Skefos J, Cummings C, Enzer K, Holiday J, Weed K, Levy E, Yuce T, Kemper T, Bauman M. Regional alterations in Purkinje Cell Density in patients with autism. PLoS ONE. 2014;9(2):e81255. 10.1371/journal.pone.0081255. 24586223 10.1371/journal.pone.0081255 PMC3933333

[60] DeRamus TP, Kana RK. Anatomical likelihood estimation meta-analysis of grey and white matter anomalies in autism spectrum disorders. NeuroImage: Clin. 2015;7:525–36. 10.1016/j.nicl.2014.11.004. 25844306 10.1016/j.nicl.2014.11.004 PMC4375647

[61] Bolduc M-E, du Plessis AJ, Sullivan N, Guizard N, Zhang X, Robertson RL, Limperopoulos C. Regional cerebellar volumes predict functional outcome in children with cerebellar malformations. Cerebellum. 2012;11(2):531–42. 10.1007/s12311-011-0312-z. 21901523 10.1007/s12311-011-0312-z

[62] Postema MC, van Rooij D, Anagnostou E, Arango C, Auzias G, Behrmann M, Filho GB, Calderoni S, Calvo R, Daly E, Deruelle C, Di Martino A, Dinstein I, Duran FLS, Durston S, Ecker C, Ehrlich S, Fair D, Fedor J, … Francks C. Altered structural brain asymmetry in autism spectrum disorder in a study of 54 datasets. Nat Commun 2019;10(1):4958. https://doi.org/10.1038/s41467-019-13005-8. 10.1038/s41467-019-13005-8 PMC6823355 31673008

[63] Olson IR, Hoffman LJ, Jobson KR, Popal HS, Wang Y. Little brain, little minds: the big role of the cerebellum in social development. Dev Cogn Neurosci. 2023;60:101238. 10.1016/j.dcn.2023.101238. 37004475 10.1016/j.dcn.2023.101238 PMC10067769

[64] D’Mello AM, Stoodley CJ. (2015). Cerebro-cerebellar circuits in autism spectrum disorder. Front Neurosci. 9. 10.3389/fnins.2015.00408. 10.3389/fnins.2015.00408 PMC4633503 26594140

[65] Fry A, Littlejohns TJ, Sudlow C, Doherty N, Adamska L, Sprosen T, Collins R, Allen NE. Comparison of Sociodemographic and Health-related characteristics of UK Biobank participants with those of the General Population. Am J Epidemiol. 2017;186(9):1026–34. 10.1093/aje/kwx246. 28641372 10.1093/aje/kwx246 PMC5860371

