# Supplementary material for: Association of polygenic scores for autism with volumetric MRI phenotypes in cerebellum and brainstem in adults
Source: Mol Autism. 2024 Aug 7;15:34. doi: 10.1186/s13229-024-00611-7 (PMC11304666; doi:10.1186/s13229-024-00611-7)
Supplement: Supplementary file 1 — Supplementary Material [file 13229_2024_611_MOESM1_ESM.docx]

Supplementary Material

**Association of Polygenic Scores for Autism with volumetric MRI phenotypes in cerebellum and brainstem in adults**

Salahuddin Mohammad^1^, Mélissa Gentreau^1^, Manon Dubol^2^, Gull Rukh^1^, ^*^Jessica Mwinyi^1^ and ^*^Helgi B. Schiöth^1^

^*^JM and HBS are joint last author

***Authors affiliation***

^1^Functional Pharmacology and Neuroscience Unit, Department of Surgical Sciences, Uppsala University, Uppsala, Sweden.

^2^Department of Women's and Children's Health, Science for Life Laboratory, Uppsala University, Sweden.

Table of Contents

[**Supplementary Figure 1:** Flowchart of UKB participants for analysis 3](#_Toc172036233)

[**Supplementary Figure 2:** Histograms of polygenic risk scores for ASD 4](#_Toc172036234)

[**Supplementary Table 1:** Number of SNPs at each *P_SNP_* threshold for the ASD polygenic risk scores 4](#_Toc172036235)

[**Supplementary Table 2:** Effects of ASD PRS on the Total Volumes (primary MRI phenotypes) 5](#_Toc172036236)

[**Supplementary Table 3:** Effects of ASD PRS on Sub-regional Volumes (Secondary MRI phenotypes) 7](#_Toc172036237)

[**Supplementary Table 4:** Effects of ASD PRS on the phenotypes after excluding individuals with ASD in UKB (*N* = 30,870) 11](#_Toc172036238)

| **Supplementary Figure 1:** Flowchart of UKB participants for analysis |
| --- |
| Participants with consent,  *N* = 502 359  Non-Caucasian participants excluded, *N* = 92 845  Self-identified as 'White British' with very similar genetic ancestry genotypes, *N* = 409 514  Participants excluded with a missing rate >0.02 on autosomes, with sex discordance, who were outliers for heterozygosity and genetically related, *N* = 72 128  UKB Total Population,  *N* = 502 371  Participants excluded who withdrawn consent from the study, *N* = 12  Quality controlled genotyped Participants for PRS,  *N* = 337 386  Participants with MRI data,  *N* = 32 301  Final sample for association analysis,  *N* = 30 895  Participants excluded with prevalent dementia, Parkinson’s disease, multiple sclerosis, epilepsy, stroke, head or neurological injury, or trauma, or other chronic neurological problems or brain tumor (cancerous and non-cancerous). *N* = 1 406  Participants excluded with missing MRI data,  *N* = 305 085 |
|  |

# **Supplementary Figure 2:** Histograms of polygenic risk scores for ASD


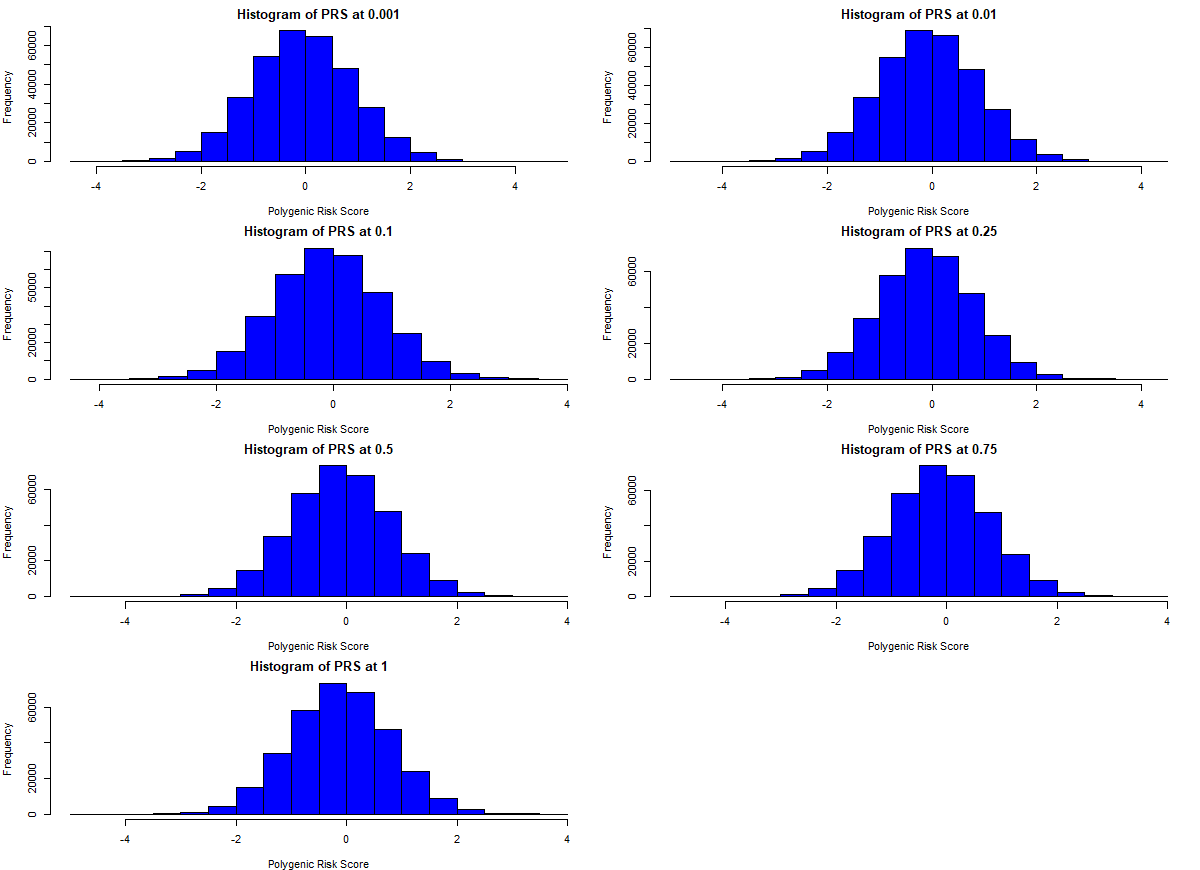


*Frequency histogram of standardized polygenic risk scores for ASD in the UK Biobank*

# **Supplementary Table 1:** Number of SNPs at each *P_SNP_* threshold for the ASD polygenic risk scores

| Threshold | SNPs (N) |
| --- | --- |
| 0.001 | 1378 |
| 0.01 | 7509 |
| 0.1 | 40798 |
| 0.25 | 77576 |
| 0.5 | 121134 |
| 0.75 | 150112 |
| 1 | 167275 |

*This table provides the number of SNPs (LD-clumped, r^2^ ≥ 0.1) for each P_SNP_ threshold for the ASD PRS*

# **Supplementary Table 2:** Effects of ASD PRS on the Total Volumes (primary MRI phenotypes)

| Phenotype | PRS | *β* | SE | *P* | *P_FDR_* | *R^2^* |
| --- | --- | --- | --- | --- | --- | --- |
| ***Total Brain Volume*** | ***0.001*** | ***-8.61E+02*** | ***3.46E+02*** | ***1.29E-02*** | ***1.71E-02*** | ***1.31E-02*** |
| Total Brain Volume | 0.01 | -1.97E+02 | 3.52E+02 | 5.75E-01 | 5.75E-01 | 6.66E-04 |
| Total Brain Volume | 0.1 | -3.40E+02 | 3.71E+02 | 3.60E-01 | 4.79E-01 | 1.78E-03 |
| Total Brain Volume | 0.5 | -6.75E+02 | 3.77E+02 | 7.34E-02 | 1.47E-01 | 6.78E-03 |
| Total Brain Volume | 0.25 | -6.32E+02 | 3.75E+02 | 9.23E-02 | 1.85E-01 | 5.99E-03 |
| Total Brain Volume | 0.75 | -6.60E+02 | 3.79E+02 | 8.17E-02 | 1.63E-01 | 6.41E-03 |
| Total Brain Volume | 1 | -6.56E+02 | 3.79E+02 | 8.35E-02 | 1.67E-01 | 6.34E-03 |
| ***CSF*** | ***0.001*** | ***3.06E+02*** | ***1.02E+02*** | ***2.80E-03*** | ***5.61E-03*** | ***2.16E-02*** |
| CSF | 0.01 | 1.91E+02 | 1.04E+02 | 6.67E-02 | 1.33E-01 | 8.14E-03 |
| CSF | 0.1 | 1.15E+02 | 1.10E+02 | 2.93E-01 | 4.79E-01 | 2.68E-03 |
| CSF | 0.5 | 1.03E+02 | 1.11E+02 | 3.56E-01 | 4.75E-01 | 2.06E-03 |
| CSF | 0.25 | 1.03E+02 | 1.11E+02 | 3.53E-01 | 4.70E-01 | 2.09E-03 |
| CSF | 0.75 | 1.17E+02 | 1.12E+02 | 2.95E-01 | 3.93E-01 | 2.66E-03 |
| CSF | 1 | 1.17E+02 | 1.12E+02 | 2.96E-01 | 3.95E-01 | 2.64E-03 |
| Grey matter | 0.001 | -8.48E+01 | 2.09E+02 | 6.86E-01 | 6.86E-01 | 2.93E-04 |
| Grey matter | 0.01 | 2.62E+02 | 2.13E+02 | 2.18E-01 | 2.91E-01 | 2.71E-03 |
| Grey matter | 0.1 | 9.61E+01 | 2.24E+02 | 6.68E-01 | 6.68E-01 | 3.29E-04 |
| Grey matter | 0.5 | -3.83E+01 | 2.28E+02 | 8.67E-01 | 8.67E-01 | 5.03E-05 |
| Grey matter | 0.25 | -2.48E+01 | 2.27E+02 | 9.13E-01 | 9.13E-01 | 2.13E-05 |
| Grey matter | 0.75 | -3.24E+01 | 2.29E+02 | 8.88E-01 | 8.88E-01 | 3.58E-05 |
| Grey matter | 1 | -3.67E+01 | 2.29E+02 | 8.73E-01 | 8.73E-01 | 4.58E-05 |
| ***White matter*** | ***0.001*** | ***-7.77E+02*** | ***2.23E+02*** | ***5.00E-04*** | ***1.96E-03*** | ***3.42E-02*** |
| White matter | 0.01 | -4.60E+02 | 2.26E+02 | 4.24E-02 | 1.33E-01 | 1.16E-02 |
| White matter | 0.1 | -4.36E+02 | 2.38E+02 | 6.76E-02 | 2.71E-01 | 9.41E-03 |
| **White matter** | **0.5** | **-6.37E+02** | **2.43E+02** | **8.70E-03** | **3.46E-02** | **1.94E-02** |
| **White matter** | **0.25** | **-6.07E+02** | **2.41E+02** | **1.19E-02** | **4.78E-02** | **1.78E-02** |
| **White matter** | **0.75** | **-6.28E+02** | **2.44E+02** | **1.01E-02** | **4.04E-02** | **1.86E-02** |
| **White matter** | **1** | **-6.19E+02** | **2.44E+02** | **1.11E-02** | **4.44E-02** | **1.82E-02** |
| Total Cerebellum | 0.001 | -3.12E+01 | 4.95E+01 | 5.28E-01 | 9.50E-01 | 7.56E-04 |
| Total Cerebellum | 0.01 | -5.46E+01 | 5.03E+01 | 2.78E-01 | 4.16E-01 | 2.24E-03 |
| Total Cerebellum | 0.1 | -8.94E+01 | 5.29E+01 | 9.13E-02 | 1.83E-01 | 5.41E-03 |
| Total Cerebellum | 0.5 | -7.38E+01 | 5.39E+01 | 1.71E-01 | 2.85E-01 | 3.56E-03 |
| Total Cerebellum | 0.25 | -8.22E+01 | 5.36E+01 | 1.25E-01 | 2.14E-01 | 4.47E-03 |
| Total Cerebellum | 0.75 | -7.47E+01 | 5.42E+01 | 1.68E-01 | 2.88E-01 | 3.61E-03 |
| Total Cerebellum | 1 | -7.61E+01 | 5.41E+01 | 1.60E-01 | 2.74E-01 | 3.75E-03 |
| **Brain Stem** | **0.001** | **-1.39E+01** | **4.20E+00** | **9.00E-04** | **5.56E-03** | **2.47E-02** |
| ***Brain Stem*** | ***0.01*** | ***-1.49E+01*** | ***4.27E+00*** | ***5.00E-04*** | ***2.97E-03*** | ***2.73E-02*** |
| **Brain Stem** | **0.1** | **-1.29E+01** | **4.50E+00** | **4.10E-03** | **2.45E-02** | **1.85E-02** |
| Brain Stem | 0.5 | -1.13E+01 | 4.58E+00 | 1.34E-02 | 8.06E-02 | 1.37E-02 |
| **Brain Stem** | **0.25** | **-1.30E+01** | **4.56E+00** | **4.50E-03** | **2.67E-02** | **1.82E-02** |
| Brain Stem | 0.75 | -1.19E+01 | 4.60E+00 | 1.00E-02 | 6.01E-02 | 1.49E-02 |
| Brain Stem | 1 | -1.17E+01 | 4.60E+00 | 1.11E-02 | 6.66E-02 | 1.45E-02 |
| Cerebellar Regions |  |  |  |  |  |  |
| ***I-IV*** | ***0.001*** | ***-9.09E+00*** | ***2.93E+00*** | ***2.00E-03*** | ***7.82E-03*** | ***2.39E-02*** |
| **I-IV** | **0.01** | **-8.42E+00** | **2.98E+00** | **4.70E-03** | **1.90E-02** | **1.98E-02** |
| I-IV | 0.1 | -7.79E+00 | 3.14E+00 | 1.31E-02 | 5.25E-02 | 1.53E-02 |
| I-IV | 0.5 | -5.77E+00 | 3.20E+00 | 7.13E-02 | 2.45E-01 | 8.09E-03 |
| I-IV | 0.25 | -6.49E+00 | 3.18E+00 | 4.12E-02 | 1.65E-01 | 1.04E-02 |
| I-IV | 0.75 | -5.59E+00 | 3.21E+00 | 8.19E-02 | 2.46E-01 | 7.53E-03 |
| I-IV | 1 | -5.57E+00 | 3.21E+00 | 8.27E-02 | 2.48E-01 | 7.49E-03 |
| V | 0.001 | -7.99E+00 | 3.60E+00 | 2.64E-02 | 7.91E-02 | 1.14E-02 |
| V | 0.01 | -7.66E+00 | 3.66E+00 | 3.62E-02 | 8.70E-02 | 1.02E-02 |
| V | 0.1 | -7.20E+00 | 3.85E+00 | 6.17E-02 | 1.83E-01 | 8.08E-03 |
| V | 0.5 | -3.24E+00 | 3.92E+00 | 4.08E-01 | 4.90E-01 | 1.58E-03 |
| V | 0.25 | -4.27E+00 | 3.90E+00 | 2.74E-01 | 3.65E-01 | 2.77E-03 |
| V | 0.75 | -3.41E+00 | 3.94E+00 | 3.88E-01 | 4.65E-01 | 1.73E-03 |
| V | 1 | -3.32E+00 | 3.94E+00 | 3.99E-01 | 4.79E-01 | 1.65E-03 |
| VI | 0.001 | 4.26E+00 | 1.00E+01 | 6.72E-01 | 9.50E-01 | 3.60E-04 |
| VI | 0.01 | 1.68E+00 | 1.02E+01 | 8.69E-01 | 9.26E-01 | 5.45E-05 |
| VI | 0.1 | -6.33E+00 | 1.08E+01 | 5.56E-01 | 5.56E-01 | 6.96E-04 |
| VI | 0.5 | -1.61E+00 | 1.09E+01 | 8.83E-01 | 8.83E-01 | 4.36E-05 |
| VI | 0.25 | -5.11E+00 | 1.09E+01 | 6.38E-01 | 6.38E-01 | 4.43E-04 |
| VI | 0.75 | -2.55E+00 | 1.10E+01 | 8.17E-01 | 8.17E-01 | 1.08E-04 |
| VI | 1 | -2.68E+00 | 1.10E+01 | 8.07E-01 | 8.07E-01 | 1.19E-04 |
| VIIb | 0.001 | -1.77E+00 | 6.71E+00 | 7.92E-01 | 9.50E-01 | 1.72E-04 |
| VIIb | 0.01 | -6.63E+00 | 6.82E+00 | 3.32E-01 | 4.42E-01 | 2.32E-03 |
| VIIb | 0.1 | -1.22E+01 | 7.18E+00 | 8.87E-02 | 1.83E-01 | 7.13E-03 |
| VIIb | 0.5 | -1.08E+01 | 7.31E+00 | 1.39E-01 | 2.85E-01 | 5.39E-03 |
| VIIb | 0.25 | -1.21E+01 | 7.27E+00 | 9.61E-02 | 1.92E-01 | 6.82E-03 |
| VIIb | 0.75 | -1.11E+01 | 7.35E+00 | 1.30E-01 | 2.88E-01 | 5.65E-03 |
| VIIb | 1 | -1.13E+01 | 7.35E+00 | 1.24E-01 | 2.71E-01 | 5.83E-03 |
| VIIIa | 0.001 | -4.01E+00 | 7.18E+00 | 5.76E-01 | 9.50E-01 | 7.12E-04 |
| VIIIa | 0.01 | -9.84E+00 | 7.30E+00 | 1.78E-01 | 3.55E-01 | 4.14E-03 |
| VIIIa | 0.1 | -1.23E+01 | 7.69E+00 | 1.09E-01 | 1.88E-01 | 5.84E-03 |
| VIIIa | 0.5 | -1.14E+01 | 7.82E+00 | 1.47E-01 | 2.85E-01 | 4.80E-03 |
| VIIIa | 0.25 | -1.31E+01 | 7.78E+00 | 9.25E-02 | 1.92E-01 | 6.45E-03 |
| VIIIa | 0.75 | -1.14E+01 | 7.86E+00 | 1.47E-01 | 2.88E-01 | 4.79E-03 |
| VIIIa | 1 | -1.17E+01 | 7.86E+00 | 1.36E-01 | 2.71E-01 | 5.08E-03 |
| VIIIb | 0.001 | -1.73E+00 | 5.28E+00 | 7.43E-01 | 9.50E-01 | 2.61E-04 |
| VIIIb | 0.01 | -5.91E+00 | 5.37E+00 | 2.71E-01 | 4.16E-01 | 2.94E-03 |
| VIIIb | 0.1 | -5.63E+00 | 5.65E+00 | 3.20E-01 | 3.83E-01 | 2.41E-03 |
| VIIIb | 0.5 | -7.54E+00 | 5.75E+00 | 1.90E-01 | 2.85E-01 | 4.17E-03 |
| VIIIb | 0.25 | -7.67E+00 | 5.72E+00 | 1.80E-01 | 2.70E-01 | 4.36E-03 |
| VIIIb | 0.75 | -7.10E+00 | 5.79E+00 | 2.20E-01 | 3.30E-01 | 3.65E-03 |
| VIIIb | 1 | -7.30E+00 | 5.78E+00 | 2.07E-01 | 3.10E-01 | 3.87E-03 |
| IX | 0.001 | -9.02E+00 | 4.50E+00 | 4.53E-02 | 1.09E-01 | 1.04E-02 |
| ***IX*** | ***0.01*** | ***-1.18E+01*** | ***4.58E+00*** | ***9.70E-03*** | ***2.90E-02*** | ***1.73E-02*** |
| IX | 0.1 | -6.98E+00 | 4.82E+00 | 1.48E-01 | 2.22E-01 | 5.43E-03 |
| IX | 0.5 | -8.54E+00 | 4.91E+00 | 8.18E-02 | 2.45E-01 | 7.85E-03 |
| IX | 0.25 | -8.60E+00 | 4.88E+00 | 7.80E-02 | 1.92E-01 | 8.05E-03 |
| IX | 0.75 | -8.77E+00 | 4.93E+00 | 7.55E-02 | 2.46E-01 | 8.19E-03 |
| IX | 1 | -8.88E+00 | 4.93E+00 | 7.17E-02 | 2.48E-01 | 8.41E-03 |
| **X** | **0.001** | **-2.66E+00** | **8.00E-01** | **9.00E-04** | **5.56E-03** | **2.47E-02** |
| ***X*** | ***0.01*** | ***-2.85E+00*** | ***8.10E-01*** | ***5.00E-04*** | ***2.97E-03*** | ***2.73E-02*** |
| **X** | **0.1** | **-2.54E+00** | **8.60E-01** | **3.10E-03** | **2.45E-02** | **1.95E-02** |
| X | 0.5 | -2.43E+00 | 8.70E-01 | 5.30E-03 | 6.41E-02 | 1.73E-02 |
| **X** | **0.25** | **-2.58E+00** | **8.70E-01** | **3.00E-03** | **2.67E-02** | **1.97E-02** |
| X | 0.75 | -2.46E+00 | 8.80E-01 | 5.00E-03 | 6.00E-02 | 1.76E-02 |
| X | 1 | -2.49E+00 | 8.80E-01 | 4.50E-03 | 5.45E-02 | 1.79E-02 |
| Crus I | 0.001 | 1.09E+00 | 1.57E+01 | 9.45E-01 | 9.82E-01 | 1.10E-05 |
| Crus I | 0.01 | 1.48E+00 | 1.60E+01 | 9.26E-01 | 9.26E-01 | 1.96E-05 |
| Crus I | 0.1 | -1.21E+01 | 1.68E+01 | 4.73E-01 | 5.16E-01 | 1.17E-03 |
| Crus I | 0.5 | -8.55E+00 | 1.71E+01 | 6.18E-01 | 6.74E-01 | 5.69E-04 |
| Crus I | 0.25 | -9.21E+00 | 1.70E+01 | 5.89E-01 | 6.38E-01 | 6.67E-04 |
| Crus I | 0.75 | -7.63E+00 | 1.72E+01 | 6.57E-01 | 7.17E-01 | 4.48E-04 |
| Crus I | 1 | -8.05E+00 | 1.72E+01 | 6.40E-01 | 6.98E-01 | 4.99E-04 |
| Crus II | 0.001 | -2.70E-01 | 1.18E+01 | 9.82E-01 | 9.82E-01 | 1.32E-06 |
| Crus II | 0.01 | -4.58E+00 | 1.20E+01 | 7.02E-01 | 8.43E-01 | 3.68E-04 |
| Crus II | 0.1 | -1.63E+01 | 1.26E+01 | 1.96E-01 | 2.62E-01 | 4.20E-03 |
| Crus II | 0.5 | -1.39E+01 | 1.29E+01 | 2.79E-01 | 3.72E-01 | 2.95E-03 |
| Crus II | 0.25 | -1.31E+01 | 1.28E+01 | 3.06E-01 | 3.67E-01 | 2.64E-03 |
| Crus II | 0.75 | -1.46E+01 | 1.29E+01 | 2.58E-01 | 3.43E-01 | 3.23E-03 |
| Crus II | 1 | -1.48E+01 | 1.29E+01 | 2.52E-01 | 3.36E-01 | 3.30E-03 |
| This table provides the result of the polygenic score analyses for the sixteen primary MRI phenotypes at seven different P-value thresholds. PRS: Polygenic risk score, *β*: regression coefﬁcient, SE: standard error, *P*: uncorrected, *P*_FDR_: false discovery rate corrected, *R^2^*: estimated variance explained by PRS in percentage (%). Significant results are bold (*P*_FDR_ < 0.05). Most predictive scores are italic and bold. | | | | | | |

# **Supplementary Table 3:** Effects of ASD PRS on Sub-regional Volumes (Secondary MRI phenotypes)

| Phenotype | PRS | *β* | SE | *P* | *P_FDR_* | *R^2^* |
| --- | --- | --- | --- | --- | --- | --- |
| Crus I (left) | 0.001 | -5.90E-01 | 8.00E+00 | 9.41E-01 | 9.60E-01 | 1.33E-05 |
| Crus I (left) | 0.01 | 3.34E+00 | 8.13E+00 | 6.81E-01 | 8.73E-01 | 4.10E-04 |
| Crus I (left) | 0.1 | -3.22E+00 | 8.56E+00 | 7.07E-01 | 7.61E-01 | 3.42E-04 |
| Crus I (left) | 0.5 | -2.06E+00 | 8.71E+00 | 8.13E-01 | 8.75E-01 | 1.36E-04 |
| Crus I (left) | 0.25 | -1.60E+00 | 8.66E+00 | 8.54E-01 | 8.53E-01 | 8.25E-05 |
| Crus I (left) | 0.75 | -1.16E+00 | 8.76E+00 | 8.95E-01 | 9.28E-01 | 4.26E-05 |
| Crus I (left) | 1 | -1.32E+00 | 8.75E+00 | 8.80E-01 | 9.12E-01 | 5.54E-05 |
| Crus I (right) | 0.001 | 1.67E+00 | 8.57E+00 | 8.46E-01 | 9.53E-01 | 8.64E-05 |
| Crus I (right) | 0.01 | -1.86E+00 | 8.71E+00 | 8.31E-01 | 9.18E-01 | 1.04E-04 |
| Crus I (right) | 0.1 | -8.83E+00 | 9.18E+00 | 3.36E-01 | 4.09E-01 | 2.11E-03 |
| Crus I (right) | 0.5 | -6.48E+00 | 9.34E+00 | 4.88E-01 | 6.21E-01 | 1.10E-03 |
| Crus I (right) | 0.25 | -7.61E+00 | 9.29E+00 | 4.13E-01 | 5.78E-01 | 1.53E-03 |
| Crus I (right) | 0.75 | -6.46E+00 | 9.39E+00 | 4.91E-01 | 6.52E-01 | 1.08E-03 |
| Crus I (right) | 1 | -6.72E+00 | 9.38E+00 | 4.74E-01 | 6.28E-01 | 1.17E-03 |
| Crus II (left) | 0.001 | -8.90E-01 | 6.15E+00 | 8.85E-01 | 9.53E-01 | 5.39E-05 |
| Crus II (left) | 0.01 | -2.89E+00 | 6.25E+00 | 6.44E-01 | 8.73E-01 | 5.50E-04 |
| Crus II (left) | 0.1 | -8.58E+00 | 6.58E+00 | 1.92E-01 | 3.59E-01 | 4.37E-03 |
| Crus II (left) | 0.5 | -7.30E+00 | 6.70E+00 | 2.76E-01 | 5.01E-01 | 3.05E-03 |
| Crus II (left) | 0.25 | -6.77E+00 | 6.66E+00 | 3.10E-01 | 4.82E-01 | 2.65E-03 |
| Crus II (left) | 0.75 | -7.75E+00 | 6.73E+00 | 2.50E-01 | 4.66E-01 | 3.40E-03 |
| Crus II (left) | 1 | -7.79E+00 | 6.73E+00 | 2.47E-01 | 4.61E-01 | 3.44E-03 |
| Crus II (right) | 0.001 | 3.00E-01 | 6.00E+00 | 9.60E-01 | 9.60E-01 | 6.33E-06 |
| Crus II (right) | 0.01 | -2.47E+00 | 6.10E+00 | 6.86E-01 | 8.73E-01 | 4.19E-04 |
| Crus II (right) | 0.1 | -7.75E+00 | 6.43E+00 | 2.28E-01 | 3.76E-01 | 3.73E-03 |
| Crus II (right) | 0.5 | -6.85E+00 | 6.54E+00 | 2.95E-01 | 5.01E-01 | 2.81E-03 |
| Crus II (right) | 0.25 | -6.69E+00 | 6.51E+00 | 3.04E-01 | 4.82E-01 | 2.71E-03 |
| Crus II (right) | 0.75 | -7.08E+00 | 6.58E+00 | 2.82E-01 | 4.66E-01 | 2.97E-03 |
| Crus II (right) | 1 | -7.19E+00 | 6.57E+00 | 2.74E-01 | 4.61E-01 | 3.07E-03 |
| IX (left) | 0.001 | -3.71E+00 | 2.02E+00 | 6.67E-02 | 1.87E-01 | 0.001 |
| IX (left) | 0.01 | -5.06E+00 | 2.06E+00 | 1.38E-02 | 5.25E-02 | 0.01 |
| IX (left) | 0.1 | -2.85E+00 | 2.16E+00 | 1.88E-01 | 3.59E-01 | 0.1 |
| IX (left) | 0.5 | -3.71E+00 | 2.20E+00 | 9.22E-02 | 3.69E-01 | 0.5 |
| IX (left) | 0.25 | -3.66E+00 | 2.19E+00 | 9.45E-02 | 2.89E-01 | 0.25 |
| IX (left) | 0.75 | -3.89E+00 | 2.21E+00 | 7.88E-02 | 3.49E-01 | 0.75 |
| IX (left) | 1 | -3.93E+00 | 2.21E+00 | 7.60E-02 | 3.20E-01 | 1 |
| IX (right) | 0.001 | -4.35E+00 | 2.24E+00 | 5.20E-02 | 1.62E-01 | 1.00E-02 |
| IX (right) | 0.01 | -5.63E+00 | 2.27E+00 | 1.33E-02 | 5.25E-02 | 1.62E-02 |
| IX (right) | 0.1 | -2.88E+00 | 2.39E+00 | 2.29E-01 | 3.76E-01 | 3.85E-03 |
| IX (right) | 0.5 | -3.68E+00 | 2.44E+00 | 1.31E-01 | 3.96E-01 | 6.04E-03 |
| IX (right) | 0.25 | -3.73E+00 | 2.42E+00 | 1.24E-01 | 2.89E-01 | 6.29E-03 |
| IX (right) | 0.75 | -3.73E+00 | 2.45E+00 | 1.28E-01 | 3.99E-01 | 6.13E-03 |
| IX (right) | 1 | -3.79E+00 | 2.45E+00 | 1.21E-01 | 3.77E-01 | 6.37E-03 |
| **I-IV (left)** | **0.001** | **-4.82E+00** | **1.50E+00** | **1.30E-03** | **3.55E-02** | **2.68E-02** |
| ***I-IV (left)*** | ***0.01*** | ***-5.07E+00*** | ***1.52E+00*** | ***8.00E-04*** | ***2.38E-02*** | ***2.87E-02*** |
| I-IV (left) | 0.1 | -4.89E+00 | 1.60E+00 | 2.20E-03 | 6.28E-02 | 2.41E-02 |
| I-IV (left) | 0.5 | -4.10E+00 | 1.63E+00 | 1.18E-02 | 1.65E-01 | 1.63E-02 |
| I-IV (left) | 0.25 | -4.50E+00 | 1.62E+00 | 5.50E-03 | 7.76E-02 | 1.99E-02 |
| I-IV (left) | 0.75 | -4.10E+00 | 1.64E+00 | 1.24E-02 | 1.73E-01 | 1.61E-02 |
| I-IV (left) | 1 | -4.08E+00 | 1.64E+00 | 1.27E-02 | 1.78E-01 | 1.60E-02 |
| I-IV (right) | 0.001 | -4.27E+00 | 1.60E+00 | 7.60E-03 | 5.31E-02 | 1.79E-02 |
| I-IV (right) | 0.01 | -3.35E+00 | 1.62E+00 | 3.92E-02 | 1.22E-01 | 1.07E-02 |
| I-IV (right) | 0.1 | -2.90E+00 | 1.71E+00 | 9.03E-02 | 2.69E-01 | 7.22E-03 |
| I-IV (right) | 0.5 | -1.66E+00 | 1.74E+00 | 3.40E-01 | 5.01E-01 | 2.29E-03 |
| I-IV (right) | 0.25 | -1.99E+00 | 1.73E+00 | 2.50E-01 | 4.38E-01 | 3.33E-03 |
| I-IV (right) | 0.75 | -1.49E+00 | 1.75E+00 | 3.94E-01 | 5.80E-01 | 1.83E-03 |
| I-IV (right) | 1 | -1.49E+00 | 1.75E+00 | 3.93E-01 | 5.79E-01 | 1.84E-03 |
| V (left) | 0.001 | -4.71E+00 | 1.94E+00 | 1.53E-02 | 7.16E-02 | 1.42E-02 |
| V (left) | 0.01 | -5.24E+00 | 1.97E+00 | 7.90E-03 | 5.25E-02 | 1.71E-02 |
| V (left) | 0.1 | -4.50E+00 | 2.08E+00 | 3.03E-02 | 1.32E-01 | 1.14E-02 |
| V (left) | 0.5 | -2.17E+00 | 2.12E+00 | 3.05E-01 | 5.01E-01 | 2.55E-03 |
| V (left) | 0.25 | -2.75E+00 | 2.10E+00 | 1.91E-01 | 3.81E-01 | 4.15E-03 |
| V (left) | 0.75 | -2.34E+00 | 2.13E+00 | 2.72E-01 | 4.66E-01 | 2.93E-03 |
| V (left) | 1 | -2.30E+00 | 2.13E+00 | 2.80E-01 | 4.61E-01 | 2.83E-03 |
| V (right) | 0.001 | -3.29E+00 | 1.89E+00 | 8.13E-02 | 2.07E-01 | 7.18E-03 |
| V (right) | 0.01 | -2.42E+00 | 1.92E+00 | 2.07E-01 | 3.63E-01 | 3.76E-03 |
| V (right) | 0.1 | -2.70E+00 | 2.02E+00 | 1.82E-01 | 3.59E-01 | 4.22E-03 |
| V (right) | 0.5 | -1.07E+00 | 2.05E+00 | 6.02E-01 | 7.15E-01 | 6.41E-04 |
| V (right) | 0.25 | -1.51E+00 | 2.04E+00 | 4.59E-01 | 5.84E-01 | 1.30E-03 |
| V (right) | 0.75 | -1.07E+00 | 2.07E+00 | 6.05E-01 | 7.37E-01 | 6.31E-04 |
| V (right) | 1 | -1.03E+00 | 2.06E+00 | 6.19E-01 | 7.53E-01 | 5.85E-04 |
| VI (left) | 0.001 | 2.38E+00 | 4.97E+00 | 6.32E-01 | 8.87E-01 | 4.75E-04 |
| VI (left) | 0.01 | 9.40E-01 | 5.05E+00 | 8.52E-01 | 9.18E-01 | 7.17E-05 |
| VI (left) | 0.1 | -3.84E+00 | 5.32E+00 | 4.71E-01 | 5.49E-01 | 1.08E-03 |
| VI (left) | 0.5 | -1.81E+00 | 5.42E+00 | 7.38E-01 | 8.26E-01 | 2.32E-04 |
| VI (left) | 0.25 | -3.34E+00 | 5.39E+00 | 5.36E-01 | 6.25E-01 | 7.94E-04 |
| VI (left) | 0.75 | -2.01E+00 | 5.45E+00 | 7.12E-01 | 7.98E-01 | 2.81E-04 |
| VI (left) | 1 | -2.04E+00 | 5.44E+00 | 7.08E-01 | 7.93E-01 | 2.90E-04 |
| VI (right) | 0.001 | 1.28E+00 | 4.87E+00 | 7.93E-01 | 9.53E-01 | 1.44E-04 |
| VI (right) | 0.01 | 4.00E-01 | 4.95E+00 | 9.35E-01 | 9.35E-01 | 1.39E-05 |
| VI (right) | 0.1 | -1.17E+00 | 5.21E+00 | 8.22E-01 | 8.52E-01 | 1.06E-04 |
| VI (right) | 0.5 | 3.30E-01 | 5.30E+00 | 9.51E-01 | 9.51E-01 | 7.93E-06 |
| VI (right) | 0.25 | -1.05E+00 | 5.27E+00 | 8.42E-01 | 8.53E-01 | 8.33E-05 |
| VI (right) | 0.75 | -2.50E-01 | 5.33E+00 | 9.63E-01 | 9.63E-01 | 4.62E-06 |
| VI (right) | 1 | -3.80E-01 | 5.33E+00 | 9.44E-01 | 9.44E-01 | 1.04E-05 |
| VIIIa (left) | 0.001 | -1.47E+00 | 3.41E+00 | 6.65E-01 | 8.87E-01 | 4.43E-04 |
| VIIIa (left) | 0.01 | -4.56E+00 | 3.46E+00 | 1.88E-01 | 3.51E-01 | 4.11E-03 |
| VIIIa (left) | 0.1 | -5.48E+00 | 3.65E+00 | 1.33E-01 | 3.37E-01 | 5.35E-03 |
| VIIIa (left) | 0.5 | -4.74E+00 | 3.71E+00 | 2.01E-01 | 4.33E-01 | 3.87E-03 |
| VIIIa (left) | 0.25 | -5.37E+00 | 3.69E+00 | 1.46E-01 | 3.14E-01 | 5.00E-03 |
| VIIIa (left) | 0.75 | -4.57E+00 | 3.73E+00 | 2.21E-01 | 4.66E-01 | 3.55E-03 |
| VIIIa (left) | 1 | -4.63E+00 | 3.73E+00 | 2.14E-01 | 4.61E-01 | 3.65E-03 |
| VIIIa (right) | 0.001 | -1.15E+00 | 3.62E+00 | 7.50E-01 | 9.53E-01 | 2.44E-04 |
| VIIIa (right) | 0.01 | -4.84E+00 | 3.68E+00 | 1.88E-01 | 3.51E-01 | 4.16E-03 |
| VIIIa (right) | 0.1 | -5.30E+00 | 3.88E+00 | 1.72E-01 | 3.59E-01 | 4.49E-03 |
| VIIIa (right) | 0.5 | -5.35E+00 | 3.94E+00 | 1.75E-01 | 4.16E-01 | 4.42E-03 |
| VIIIa (right) | 0.25 | -6.26E+00 | 3.92E+00 | 1.11E-01 | 2.89E-01 | 6.12E-03 |
| VIIIa (right) | 0.75 | -5.64E+00 | 3.97E+00 | 1.55E-01 | 4.07E-01 | 4.87E-03 |
| VIIIa (right) | 1 | -5.88E+00 | 3.96E+00 | 1.38E-01 | 3.79E-01 | 5.29E-03 |
| VIIIb (left) | 0.001 | 1.65E+00 | 2.58E+00 | 5.23E-01 | 8.13E-01 | 1.02E-03 |
| VIIIb (left) | 0.01 | -1.24E+00 | 2.62E+00 | 6.36E-01 | 8.73E-01 | 5.58E-04 |
| VIIIb (left) | 0.1 | -1.52E+00 | 2.76E+00 | 5.82E-01 | 6.52E-01 | 7.55E-04 |
| VIIIb (left) | 0.5 | -2.08E+00 | 2.81E+00 | 4.60E-01 | 6.21E-01 | 1.36E-03 |
| VIIIb (left) | 0.25 | -1.85E+00 | 2.80E+00 | 5.07E-01 | 6.18E-01 | 1.09E-03 |
| VIIIb (left) | 0.75 | -1.85E+00 | 2.83E+00 | 5.13E-01 | 6.52E-01 | 1.07E-03 |
| VIIIb (left) | 1 | -1.93E+00 | 2.83E+00 | 4.94E-01 | 6.28E-01 | 1.17E-03 |
| VIIIb (right) | 0.001 | -2.13E+00 | 2.81E+00 | 4.48E-01 | 8.13E-01 | 1.48E-03 |
| VIIIb (right) | 0.01 | -3.94E+00 | 2.85E+00 | 1.67E-01 | 3.51E-01 | 4.90E-03 |
| VIIIb (right) | 0.1 | -3.15E+00 | 3.00E+00 | 2.94E-01 | 4.09E-01 | 2.83E-03 |
| VIIIb (right) | 0.5 | -4.49E+00 | 3.06E+00 | 1.41E-01 | 3.96E-01 | 5.55E-03 |
| VIIIb (right) | 0.25 | -4.75E+00 | 3.04E+00 | 1.19E-01 | 2.89E-01 | 6.25E-03 |
| VIIIb (right) | 0.75 | -4.32E+00 | 3.07E+00 | 1.60E-01 | 4.07E-01 | 5.07E-03 |
| VIIIb (right) | 1 | -4.43E+00 | 3.07E+00 | 1.49E-01 | 3.79E-01 | 5.34E-03 |
| VIIb (left) | 0.001 | -2.19E+00 | 3.37E+00 | 5.16E-01 | 8.13E-01 | 1.06E-03 |
| VIIb (left) | 0.01 | -4.54E+00 | 3.43E+00 | 1.86E-01 | 3.51E-01 | 4.42E-03 |
| VIIb (left) | 0.1 | -7.80E+00 | 3.61E+00 | 3.08E-02 | 1.32E-01 | 1.18E-02 |
| VIIb (left) | 0.5 | -6.89E+00 | 3.68E+00 | 6.08E-02 | 3.41E-01 | 8.86E-03 |
| VIIb (left) | 0.25 | -7.45E+00 | 3.66E+00 | 4.16E-02 | 2.13E-01 | 1.05E-02 |
| VIIb (left) | 0.75 | -6.77E+00 | 3.70E+00 | 6.70E-02 | 3.49E-01 | 8.46E-03 |
| VIIb (left) | 1 | -6.83E+00 | 3.69E+00 | 6.43E-02 | 3.20E-01 | 8.63E-03 |
| VIIb (right) | 0.001 | 5.20E-01 | 3.57E+00 | 8.85E-01 | 9.53E-01 | 5.26E-05 |
| VIIb (right) | 0.01 | -2.14E+00 | 3.63E+00 | 5.56E-01 | 8.73E-01 | 8.71E-04 |
| VIIb (right) | 0.1 | -4.27E+00 | 3.82E+00 | 2.64E-01 | 4.09E-01 | 3.13E-03 |
| VIIb (right) | 0.5 | -3.77E+00 | 3.89E+00 | 3.32E-01 | 5.01E-01 | 2.36E-03 |
| VIIb (right) | 0.25 | -4.50E+00 | 3.87E+00 | 2.45E-01 | 4.38E-01 | 3.40E-03 |
| VIIb (right) | 0.75 | -4.20E+00 | 3.91E+00 | 2.83E-01 | 4.66E-01 | 2.90E-03 |
| VIIb (right) | 1 | -4.31E+00 | 3.91E+00 | 2.70E-01 | 4.61E-01 | 3.06E-03 |
| Crus I (vermis) | 0.001 | 1.00E-02 | 1.00E-02 | 1.29E-01 | 2.77E-01 | 7.34E-03 |
| Crus I (vermis) | 0.01 | 0.00E+00 | 1.00E-02 | 8.89E-01 | 9.21E-01 | 6.24E-05 |
| Crus I (vermis) | 0.1 | -1.00E-02 | 1.00E-02 | 3.25E-01 | 4.09E-01 | 3.08E-03 |
| Crus I (vermis) | 0.5 | -1.00E-02 | 1.00E-02 | 4.88E-01 | 6.21E-01 | 1.53E-03 |
| Crus I (vermis) | 0.25 | 0.00E+00 | 1.00E-02 | 6.21E-01 | 6.68E-01 | 7.78E-04 |
| Crus I (vermis) | 0.75 | -1.00E-02 | 1.00E-02 | 4.64E-01 | 6.50E-01 | 1.70E-03 |
| Crus I (vermis) | 1 | -1.00E-02 | 1.00E-02 | 4.85E-01 | 6.28E-01 | 1.55E-03 |
| Crus II (vermis) | 0.001 | 3.20E-01 | 4.30E-01 | 4.50E-01 | 8.13E-01 | 1.71E-03 |
| Crus II (vermis) | 0.01 | 7.70E-01 | 4.30E-01 | 7.49E-02 | 2.10E-01 | 9.53E-03 |
| Crus II (vermis) | 0.1 | 1.00E-02 | 4.60E-01 | 9.81E-01 | 9.81E-01 | 1.77E-06 |
| Crus II (vermis) | 0.5 | 2.40E-01 | 4.60E-01 | 6.13E-01 | 7.15E-01 | 7.70E-04 |
| Crus II (vermis) | 0.25 | 3.60E-01 | 4.60E-01 | 4.42E-01 | 5.84E-01 | 1.78E-03 |
| Crus II (vermis) | 0.75 | 1.90E-01 | 4.70E-01 | 6.82E-01 | 7.96E-01 | 5.04E-04 |
| Crus II (vermis) | 1 | 1.90E-01 | 4.70E-01 | 6.79E-01 | 7.92E-01 | 5.14E-04 |
| IX (vermis) | 0.001 | -9.60E-01 | 4.70E-01 | 3.94E-02 | 1.38E-01 | 1.11E-02 |
| IX (vermis) | 0.01 | -1.16E+00 | 4.80E-01 | 1.50E-02 | 5.25E-02 | 1.55E-02 |
| IX (vermis) | 0.1 | -1.24E+00 | 5.00E-01 | 1.30E-02 | 1.32E-01 | 1.62E-02 |
| IX (vermis) | 0.5 | -1.15E+00 | 5.10E-01 | 2.34E-02 | 2.18E-01 | 1.35E-02 |
| IX (vermis) | 0.25 | -1.20E+00 | 5.10E-01 | 1.74E-02 | 1.62E-01 | 1.48E-02 |
| IX (vermis) | 0.75 | -1.15E+00 | 5.10E-01 | 2.48E-02 | 2.31E-01 | 1.32E-02 |
| IX (vermis) | 1 | -1.16E+00 | 5.10E-01 | 2.36E-02 | 2.21E-01 | 1.34E-02 |
| VI (vermis) | 0.001 | 6.00E-01 | 1.28E+00 | 6.41E-01 | 8.87E-01 | 5.95E-04 |
| VI (vermis) | 0.01 | 3.40E-01 | 1.30E+00 | 7.94E-01 | 9.18E-01 | 1.87E-04 |
| VI (vermis) | 0.1 | -1.32E+00 | 1.37E+00 | 3.33E-01 | 4.09E-01 | 2.57E-03 |
| VI (vermis) | 0.5 | -1.30E-01 | 1.39E+00 | 9.28E-01 | 9.51E-01 | 2.23E-05 |
| VI (vermis) | 0.25 | -7.20E-01 | 1.38E+00 | 6.01E-01 | 6.68E-01 | 7.48E-04 |
| VI (vermis) | 0.75 | -2.90E-01 | 1.40E+00 | 8.35E-01 | 8.99E-01 | 1.19E-04 |
| VI (vermis) | 1 | -2.70E-01 | 1.40E+00 | 8.47E-01 | 9.12E-01 | 1.01E-04 |
| VIIIa (vermis) | 0.001 | -1.38E+00 | 8.60E-01 | 1.06E-01 | 2.47E-01 | 6.51E-03 |
| VIIIa (vermis) | 0.01 | -4.40E-01 | 8.70E-01 | 6.15E-01 | 8.73E-01 | 6.30E-04 |
| VIIIa (vermis) | 0.1 | -1.52E+00 | 9.20E-01 | 9.61E-02 | 2.69E-01 | 6.90E-03 |
| VIIIa (vermis) | 0.5 | -1.25E+00 | 9.30E-01 | 1.78E-01 | 4.16E-01 | 4.51E-03 |
| VIIIa (vermis) | 0.25 | -1.46E+00 | 9.30E-01 | 1.14E-01 | 2.89E-01 | 6.21E-03 |
| VIIIa (vermis) | 0.75 | -1.19E+00 | 9.40E-01 | 2.06E-01 | 4.66E-01 | 3.99E-03 |
| VIIIa (vermis) | 1 | -1.22E+00 | 9.40E-01 | 1.92E-01 | 4.47E-01 | 4.25E-03 |
| ***VIIIb (vermis)*** | ***0.001*** | ***-1.25E+00*** | ***4.40E-01*** | ***4.80E-03*** | ***4.49E-02*** | ***2.08E-02*** |
| VIIIb (vermis) | 0.01 | -7.30E-01 | 4.50E-01 | 1.07E-01 | 2.72E-01 | 6.79E-03 |
| VIIIb (vermis) | 0.1 | -9.50E-01 | 4.80E-01 | 4.51E-02 | 1.58E-01 | 1.05E-02 |
| VIIIb (vermis) | 0.5 | -9.60E-01 | 4.80E-01 | 4.66E-02 | 3.26E-01 | 1.03E-02 |
| VIIIb (vermis) | 0.25 | -1.07E+00 | 4.80E-01 | 2.59E-02 | 1.81E-01 | 1.30E-02 |
| VIIIb (vermis) | 0.75 | -9.30E-01 | 4.90E-01 | 5.73E-02 | 3.49E-01 | 9.44E-03 |
| VIIIb (vermis) | 1 | -9.30E-01 | 4.90E-01 | 5.56E-02 | 3.20E-01 | 9.57E-03 |
| VIIb (vermis) | 0.001 | -1.00E-01 | 1.50E-01 | 4.98E-01 | 8.13E-01 | 1.27E-03 |
| VIIb (vermis) | 0.01 | 5.00E-02 | 1.50E-01 | 7.42E-01 | 9.03E-01 | 2.99E-04 |
| VIIb (vermis) | 0.1 | -1.70E-01 | 1.60E-01 | 3.00E-01 | 4.09E-01 | 2.97E-03 |
| VIIb (vermis) | 0.5 | -1.60E-01 | 1.60E-01 | 3.31E-01 | 5.01E-01 | 2.62E-03 |
| VIIb (vermis) | 0.25 | -1.50E-01 | 1.60E-01 | 3.41E-01 | 5.03E-01 | 2.50E-03 |
| VIIb (vermis) | 0.75 | -1.70E-01 | 1.60E-01 | 3.07E-01 | 4.78E-01 | 2.88E-03 |
| VIIb (vermis) | 1 | -1.60E-01 | 1.60E-01 | 3.23E-01 | 5.02E-01 | 2.70E-03 |
| ***X (vermis)*** | ***0.001*** | ***-6.60E-01*** | ***2.30E-01*** | ***4.50E-03*** | ***4.49E-02*** | ***2.19E-02*** |
| X (vermis) | 0.01 | -6.30E-01 | 2.30E-01 | 7.00E-03 | 5.25E-02 | 1.97E-02 |
| X (vermis) | 0.1 | -5.30E-01 | 2.50E-01 | 3.30E-02 | 1.32E-01 | 1.23E-02 |
| X (vermis) | 0.5 | -3.80E-01 | 2.50E-01 | 1.33E-01 | 3.96E-01 | 6.10E-03 |
| X (vermis) | 0.25 | -4.50E-01 | 2.50E-01 | 7.50E-02 | 2.89E-01 | 8.58E-03 |
| X (vermis) | 0.75 | -4.10E-01 | 2.50E-01 | 1.03E-01 | 3.59E-01 | 7.22E-03 |
| X (vermis) | 1 | -4.30E-01 | 2.50E-01 | 9.15E-02 | 3.20E-01 | 7.71E-03 |
| X (left) | 0.001 | -1.06E+00 | 4.10E-01 | 9.60E-03 | 5.40E-02 | 1.62E-02 |
| X (left) | 0.01 | -1.06E+00 | 4.20E-01 | 1.09E-02 | 5.25E-02 | 1.57E-02 |
| X (left) | 0.1 | -9.60E-01 | 4.40E-01 | 2.92E-02 | 1.32E-01 | 1.15E-02 |
| X (left) | 0.5 | -7.90E-01 | 4.50E-01 | 7.60E-02 | 3.55E-01 | 7.61E-03 |
| X (left) | 0.25 | -8.90E-01 | 4.50E-01 | 4.55E-02 | 2.13E-01 | 9.67E-03 |
| X (left) | 0.75 | -7.70E-01 | 4.50E-01 | 8.72E-02 | 3.49E-01 | 7.07E-03 |
| X (left) | 1 | -7.70E-01 | 4.50E-01 | 8.62E-02 | 3.20E-01 | 7.12E-03 |
| X (right) | 0.001 | -9.40E-01 | 4.10E-01 | 2.22E-02 | 8.89E-02 | 1.27E-02 |
| X (right) | 0.01 | -1.15E+00 | 4.20E-01 | 6.10E-03 | 5.25E-02 | 1.83E-02 |
| X (right) | 0.1 | -1.05E+00 | 4.40E-01 | 1.77E-02 | 1.32E-01 | 1.37E-02 |
| X (right) | 0.5 | -1.26E+00 | 4.50E-01 | 5.20E-03 | 1.46E-01 | 1.90E-02 |
| X (right) | 0.25 | -1.24E+00 | 4.50E-01 | 5.50E-03 | 7.76E-02 | 1.87E-02 |
| X (right) | 0.75 | -1.28E+00 | 4.50E-01 | 4.70E-03 | 1.33E-01 | 1.94E-02 |
| X (right) | 1 | -1.29E+00 | 4.50E-01 | 4.40E-03 | 1.23E-01 | 1.98E-02 |
| This table provides the result of the polygenic score analyses for the twenty-eight secondary MRI phenotypes at seven different P-value thresholds. PRS: Polygenic risk score, *β*: regression coefﬁcient, SE: standard error, *P*: uncorrected, *P*_FDR_: false discovery rate corrected, *R^2^*: estimated variance explained by PRS in percentage (%). Significant results are bold (*P*_FDR_ < 0.05). Most predictive scores are italic and bold. | | | | | | |

# **Supplementary Table 4:** Effects of ASD PRS on the phenotypes after excluding individuals with ASD in UKB (*N* = 30,870)

| **Total Volumes** | | | | | | |
| --- | --- | --- | --- | --- | --- | --- |
| Phenotype | PRS | *β* | SE | *P* | *P_FDR_* | *R^2^* |
| ***Total Brain Volume*** | ***0.001*** | ***-8.61E+02*** | ***3.46E+02*** | ***1.29E-02*** | ***1.71E-02*** | ***1.31E-02*** |
| Total Brain Volume | 0.01 | -1.97E+02 | 3.52E+02 | 5.75E-01 | 5.75E-01 | 6.66E-04 |
| Total Brain Volume | 0.1 | -3.40E+02 | 3.71E+02 | 3.60E-01 | 4.79E-01 | 1.78E-03 |
| Total Brain Volume | 0.5 | -6.75E+02 | 3.77E+02 | 7.34E-02 | 1.47E-01 | 6.78E-03 |
| Total Brain Volume | 0.25 | -6.32E+02 | 3.75E+02 | 9.23E-02 | 1.85E-01 | 5.99E-03 |
| Total Brain Volume | 0.75 | -6.60E+02 | 3.79E+02 | 8.17E-02 | 1.63E-01 | 6.41E-03 |
| Total Brain Volume | 1 | -6.56E+02 | 3.79E+02 | 8.35E-02 | 1.67E-01 | 6.34E-03 |
| ***CSF*** | ***0.001*** | ***3.06E+02*** | ***1.02E+02*** | ***2.80E-03*** | ***5.61E-03*** | ***2.16E-02*** |
| CSF | 0.01 | 1.91E+02 | 1.04E+02 | 6.67E-02 | 1.33E-01 | 8.14E-03 |
| CSF | 0.1 | 1.15E+02 | 1.10E+02 | 2.93E-01 | 4.79E-01 | 2.68E-03 |
| CSF | 0.5 | 1.03E+02 | 1.11E+02 | 3.56E-01 | 4.75E-01 | 2.06E-03 |
| CSF | 0.25 | 1.03E+02 | 1.11E+02 | 3.53E-01 | 4.70E-01 | 2.09E-03 |
| CSF | 0.75 | 1.17E+02 | 1.12E+02 | 2.95E-01 | 3.93E-01 | 2.66E-03 |
| CSF | 1 | 1.17E+02 | 1.12E+02 | 2.96E-01 | 3.95E-01 | 2.64E-03 |
| ***White matter*** | ***0.001*** | ***-7.77E+02*** | ***2.23E+02*** | ***5.00E-04*** | ***1.96E-03*** | ***3.42E-02*** |
| White matter | 0.01 | -4.60E+02 | 2.26E+02 | 4.24E-02 | 1.33E-01 | 1.16E-02 |
| White matter | 0.1 | -4.36E+02 | 2.38E+02 | 6.76E-02 | 2.71E-01 | 9.41E-03 |
| **White matter** | **0.5** | **-6.37E+02** | **2.43E+02** | **8.70E-03** | **3.46E-02** | **1.94E-02** |
| **White matter** | **0.25** | **-6.07E+02** | **2.41E+02** | **1.19E-02** | **4.78E-02** | **1.78E-02** |
| **White matter** | **0.75** | **-6.28E+02** | **2.44E+02** | **1.01E-02** | **4.04E-02** | **1.86E-02** |
| **White matter** | **1** | **-6.19E+02** | **2.44E+02** | **1.11E-02** | **4.44E-02** | **1.82E-02** |
| **Brain Stem** | **0.001** | **-1.39E+01** | **4.20E+00** | **9.00E-04** | **5.56E-03** | **2.47E-02** |
| ***Brain Stem*** | ***0.01*** | ***-1.49E+01*** | ***4.27E+00*** | ***5.00E-04*** | ***2.97E-03*** | ***2.73E-02*** |
| **Brain Stem** | **0.1** | **-1.29E+01** | **4.50E+00** | **4.10E-03** | **2.45E-02** | **1.85E-02** |
| Brain Stem | 0.5 | -1.13E+01 | 4.58E+00 | 1.34E-02 | 8.06E-02 | 1.37E-02 |
| **Brain Stem** | **0.25** | **-1.30E+01** | **4.56E+00** | **4.50E-03** | **2.67E-02** | **1.82E-02** |
| Brain Stem | 0.75 | -1.19E+01 | 4.60E+00 | 1.00E-02 | 6.01E-02 | 1.49E-02 |
| Brain Stem | 1 | -1.17E+01 | 4.60E+00 | 1.11E-02 | 6.66E-02 | 1.45E-02 |
| Cerebellar Regions | | | | | | |
| ***I-IV*** | ***0.001*** | ***-9.09E+00*** | ***2.93E+00*** | ***2.00E-03*** | ***7.82E-03*** | ***2.39E-02*** |
| **I-IV** | **0.01** | **-8.42E+00** | **2.98E+00** | **4.70E-03** | **1.90E-02** | **1.98E-02** |
| I-IV | 0.1 | -7.79E+00 | 3.14E+00 | 1.31E-02 | 5.25E-02 | 1.53E-02 |
| I-IV | 0.5 | -5.77E+00 | 3.20E+00 | 7.13E-02 | 2.45E-01 | 8.09E-03 |
| I-IV | 0.25 | -6.49E+00 | 3.18E+00 | 4.12E-02 | 1.65E-01 | 1.04E-02 |
| I-IV | 0.75 | -5.59E+00 | 3.21E+00 | 8.19E-02 | 2.46E-01 | 7.53E-03 |
| I-IV | 1 | -5.57E+00 | 3.21E+00 | 8.27E-02 | 2.48E-01 | 7.49E-03 |
| IX | 0.001 | -9.02E+00 | 4.50E+00 | 4.53E-02 | 1.09E-01 | 1.04E-02 |
| ***IX*** | ***0.01*** | ***-1.18E+01*** | ***4.58E+00*** | ***9.70E-03*** | ***2.90E-02*** | ***1.73E-02*** |
| IX | 0.1 | -6.98E+00 | 4.82E+00 | 1.48E-01 | 2.22E-01 | 5.43E-03 |
| IX | 0.5 | -8.54E+00 | 4.91E+00 | 8.18E-02 | 2.45E-01 | 7.85E-03 |
| IX | 0.25 | -8.60E+00 | 4.88E+00 | 7.80E-02 | 1.92E-01 | 8.05E-03 |
| IX | 0.75 | -8.77E+00 | 4.93E+00 | 7.55E-02 | 2.46E-01 | 8.19E-03 |
| IX | 1 | -8.88E+00 | 4.93E+00 | 7.17E-02 | 2.48E-01 | 8.41E-03 |
| **X** | **0.001** | **-2.66E+00** | **8.00E-01** | **9.00E-04** | **5.56E-03** | **2.47E-02** |
| ***X*** | ***0.01*** | ***-2.85E+00*** | ***8.10E-01*** | ***5.00E-04*** | ***2.97E-03*** | ***2.73E-02*** |
| **X** | **0.1** | **-2.54E+00** | **8.60E-01** | **3.10E-03** | **2.45E-02** | **1.95E-02** |
| X | 0.5 | -2.43E+00 | 8.70E-01 | 5.30E-03 | 6.41E-02 | 1.73E-02 |
| **X** | **0.25** | **-2.58E+00** | **8.70E-01** | **3.00E-03** | **2.67E-02** | **1.97E-02** |
| X | 0.75 | -2.46E+00 | 8.80E-01 | 5.00E-03 | 6.00E-02 | 1.76E-02 |
| X | 1 | -2.49E+00 | 8.80E-01 | 4.50E-03 | 5.45E-02 | 1.79E-02 |
| **Sub-regional Volumes** | | | | | | |
| Phenotype | PRS | *β* | SE | *P* | *P_FDR_* | *R^2^* |
| **I-IV (left)** | **0.001** | **-4.82E+00** | **1.50E+00** | **1.30E-03** | **3.55E-02** | **2.68E-02** |
| ***I-IV (left)*** | ***0.01*** | ***-5.07E+00*** | ***1.52E+00*** | ***8.00E-04*** | ***2.38E-02*** | ***2.87E-02*** |
| I-IV (left) | 0.1 | -4.89E+00 | 1.60E+00 | 2.20E-03 | 6.28E-02 | 2.41E-02 |
| I-IV (left) | 0.5 | -4.10E+00 | 1.63E+00 | 1.18E-02 | 1.65E-01 | 1.63E-02 |
| I-IV (left) | 0.25 | -4.50E+00 | 1.62E+00 | 5.50E-03 | 7.76E-02 | 1.99E-02 |
| I-IV (left) | 0.75 | -4.10E+00 | 1.64E+00 | 1.24E-02 | 1.73E-01 | 1.61E-02 |
| I-IV (left) | 1 | -4.08E+00 | 1.64E+00 | 1.27E-02 | 1.78E-01 | 1.60E-02 |
| ***VIIIb (vermis)*** | ***0.001*** | ***-1.25E+00*** | ***4.40E-01*** | ***4.80E-03*** | ***4.49E-02*** | ***2.08E-02*** |
| VIIIb (vermis) | 0.01 | -7.30E-01 | 4.50E-01 | 1.07E-01 | 2.72E-01 | 6.79E-03 |
| VIIIb (vermis) | 0.1 | -9.50E-01 | 4.80E-01 | 4.51E-02 | 1.58E-01 | 1.05E-02 |
| VIIIb (vermis) | 0.5 | -9.60E-01 | 4.80E-01 | 4.66E-02 | 3.26E-01 | 1.03E-02 |
| VIIIb (vermis) | 0.25 | -1.07E+00 | 4.80E-01 | 2.59E-02 | 1.81E-01 | 1.30E-02 |
| VIIIb (vermis) | 0.75 | -9.30E-01 | 4.90E-01 | 5.73E-02 | 3.49E-01 | 9.44E-03 |
| VIIIb (vermis) | 1 | -9.30E-01 | 4.90E-01 | 5.56E-02 | 3.20E-01 | 9.57E-03 |
| ***X (vermis)*** | ***0.001*** | ***-6.60E-01*** | ***2.30E-01*** | ***4.50E-03*** | ***4.49E-02*** | ***2.19E-02*** |
| X (vermis) | 0.01 | -6.30E-01 | 2.30E-01 | 7.00E-03 | 5.25E-02 | 1.97E-02 |
| X (vermis) | 0.1 | -5.30E-01 | 2.50E-01 | 3.30E-02 | 1.32E-01 | 1.23E-02 |
| X (vermis) | 0.5 | -3.80E-01 | 2.50E-01 | 1.33E-01 | 3.96E-01 | 6.10E-03 |
| X (vermis) | 0.25 | -4.50E-01 | 2.50E-01 | 7.50E-02 | 2.89E-01 | 8.58E-03 |
| X (vermis) | 0.75 | -4.10E-01 | 2.50E-01 | 1.03E-01 | 3.59E-01 | 7.22E-03 |
| X (vermis) | 1 | -4.30E-01 | 2.50E-01 | 9.15E-02 | 3.20E-01 | 7.71E-03 |
| This table provides the result of the polygenic score analyses for the MRI phenotypes of interest (observed significant in the study) in the UK Biobank after excluding individuals with self-reported physician-posed ASD diagnosis (UKB Data-field 20544) at seven different P-value thresholds. After quality control of the study, exclusion for diagnosed diseases (see ‘Method’ section) and excluding the participants with self-reported physician-posed ASD diagnosis in UK Biobank, the number of participants with MRI data were *(N)*= 30,870 and were included in this analysis. PRS: Polygenic risk score, *β*: regression coefﬁcient, SE: standard error, *P*: uncorrected, *P*_FDR_: false discovery rate corrected, *R^2^*: estimated variance explained by PRS in percentage (%). Significant results are bold (*P*_FDR_ < 0.05). Most predictive scores are italic and bold. | | | | | | |
